# A conceptual–perceptual distinctiveness processing account of the superior recognition memory of pictures over environmental sounds

**DOI:** 10.1177/17470218231202986

**Published:** 2023-10-17

**Authors:** Fahad N Ahmad, Savannah Tremblay, Michael D Karkuszewski, Marium Alvi, William E Hockley

**Affiliations:** 1 Department of Psychology, Wilfrid Laurier University, Waterloo, ON, Canada; 2 Department of Psychology, University of Toronto, Toronto, ON, Canada; 3 Rotman Research Institute at Baycrest, Toronto, ON, Canada; 4 Department of Psychology, Western University, London, ON, Canada; 5 Department of Psychology, York University, Toronto, ON, Canada

**Keywords:** Conceptual–perceptual distinctiveness, recognition memory, gist-based representation, verbatim-based representation, similarity

## Abstract

Researchers have proposed a coarser or gist-based representation for sounds, whereas a more verbatim-based representation is retrieved from long-term memory to account for higher recognition performance for pictures. This study examined the mechanism for the recognition advantage for pictures. In Experiment 1A, pictures and sounds were presented in separate trials in a mixed list during the study phase and participants showed in a yes–no test, a higher proportion of correct responses for targets, exemplar foils categorically related to the target, and novel foils for pictures compared with sounds. In Experiment 1B, the picture recognition advantage was replicated in a two-alternative forced-choice test for the novel and exemplar foil conditions. For Experiment 2A, even when verbal labels (i.e., written labels) were presented for sounds during the study phase, a recognition advantage for pictures was shown for both targets and exemplar foils. Experiment 2B showed that the presence of written labels for sounds, during both the study and test phases did not eliminate the advantage of recognition of pictures in terms of correct rejection of exemplar foils. Finally, in two additional experiments, we examined whether the degree of similarity within pictures and sounds could account for the recognition advantage of pictures. The mean similarity rating for pictures was higher than the mean similarity rating for sounds in the exemplar test condition, whereas mean similarity rating for sounds was higher than pictures in the novel test condition. These results pose a challenge for some versions of distinctiveness accounts of the picture superiority effect. We propose a conceptual–perceptual distinctiveness processing account of recognition memory for pictures and sounds.

Several studies have shown that recognition memory is typically better for pictures compared with words in tests of item and associative recognition (Hockley, 2008; Potter & Faulconer, 1975; Snodgrass & McClure, 1975), an advantage termed the picture superiority effect (PSE). More recently, researchers have shown that long-term recognition memory for pictures is also superior to natural or environmental sounds (Bigelow & Poremba, 2014; Cohen et al., 2009, 2011; Gloede et al., 2017; Gloede & Gregg, 2019; Kassim et al., 2018). Participants in the second experiment by Cohen et al. (2009) were either presented with pictures or sounds during the study phase and were tested on their memory for the sounds or pictures in a yes–no recognition test. Moreover, two additional conditions were included to improve the participant’s encoding of sounds. In one condition, the sounds were paired with verbal descriptions as in verbal labels (i.e., written labels) during the study phase and in the other condition, the sounds were paired with pictures. Recognition memory for pictures was found to be significantly higher than sounds in all conditions.

In a follow-up study with a similar paradigm, Cohen et al. (2011) found that musicians showed higher recognition memory for sounds compared with non-musicians; however, both groups showed a recognition advantage for pictures over sounds. Cohen et al. (2011) argued that there was a fundamental difference between visual and auditory stimuli, or visual and auditory processing, with visual always being superior, but they did not suggest what such a difference might be. In a related study, Bigelow and Poremba (2014) found recognition memory for auditory sounds to be inferior to both pictures and tactile stimuli. In their second experiment, 82 participants were tested on their recognition memory of sounds, pictures, and tactile stimuli. The recognition memory phase consisted of a study and a test block for each modality. The sounds comprised sound recordings of easily recognisable, everyday events (e.g., dog barking). Instead of pictures, videos were chosen to equate visual stimuli to sounds in being dynamic, as in the stimulus information unfolded over time. Auditory recognition accuracy was significantly lower both after immediate test and after one day. Bigelow and Poremba (2014) concluded human beings have inferior retention capabilities for auditory events and memory is modality dependent, due to a consequence of differences in neural pathways in which memoranda are processed.

Interestingly, the superiority of visual memory is maintained even with training of participants with studied sounds (Gloede et al., 2017). Gloede et al. (2017) showed that increasing the amount of auditory training did not reduce superior recognition memory for pictures compared with sounds. In their first experiment, 16 participants were first presented with 64 sounds that had been used by Cohen et al. (2009) and participants were then tested on their recognition memory of the sounds using an old/new recognition memory test. The second task was similar to the first, but instead pictures were presented. After participants had completed the two tasks, there was an auditory training task, in which participants were presented with discrimination trials after which there was a second recognition memory test with pictures and sounds. Gloede et al. (2017) found that recognition performance for pictures was superior to recognition of sounds, before and after auditory training. Gloede et al. (2017) concluded experience with auditory sounds does not eliminate the auditory memory inferiority and as their Experiment 4 showed, the memory representation for pictures is inherently different from that of sounds, which could account for the auditory memory inferiority.

One theory that could account for this fundamental difference between the memory representation of pictures and sounds as shown in auditory memory inferiority is Fuzzy-trace theory (Brainerd & Reyna, 2002, 2004). According to fuzzy-trace theory, memory performance is dependent on the retrieval of gist and verbatim traces. As Ahmad, Moscovitch and Hockley, (2017) noted, gist traces are episodic representations of concepts (meanings, relations, patterns). For example, a person could rely on gist information stored in long-term memory (LTM) to distinguish a studied picture of a bell from a new picture of a soccer ball. The new item belongs to a different semantic category and the new item’s perceptual or verbatim details differ from those of the studied item. In contrast, verbatim traces are episodically instantiated representations of surface forms of experienced items. Thus, the specific surface features of an item are reflected in verbatim representations, whereas information at the level of meaning is represented in gist representations (Ahmad et al., 2017). For example, a person would rely on verbatim information stored in LTM to distinguish a studied picture of a beach ball from a perceptually similar but different beach ball. Verbatim information present in detailed visual memories would also be necessary to distinguish a beach scene from a perceptually similar but different beach scene.

In relevance to this study in which recognition memory was tested for pictures and sounds, there were two alternative predictions according to fuzzy-trace theory that could be tested. These predictions involve comparing recognition memory performance for targets, novel foils, and exemplar foils. To determine whether recognition memory is higher for pictures compared with sounds due to more retrieval of verbatim information, one would compare hit rate for studied items and correct rejection rate for similar or exemplar foils. In contrast, to investigate whether recognition memory is higher for pictures compared with sounds due to more retrieval of gist information, one would compare hit rate for studied and correct rejection rate for non-studied items or novel foils. Consequently, fuzzy-trace theory offers two distinct predictions. One prediction being that pictures would be better remembered than sounds because pictures tend to have more perceptual details associated with verbatim information stored in LTM compared with sounds This would result in higher recognition memory for pictures, evidenced by a greater accuracy in correct rejection of exemplar foil pictures and higher hit rate for target pictures compared with sounds. Alternatively, there would be higher hit rate for targets and higher correct rejection rate for exemplar foil, and novel foil pictures than sounds due to a greater amount of both gist and verbatim information stored in LTM for pictures compared with sounds. As a result, recognition memory for all types of test probes would be higher for pictures in comparison with sounds.

Support for a fuzzy-trace theory account of the superior recognition memory for pictures compared with sounds was provided by Gloede and Gregg (2019). They examined further the extent of the superiority of recognition memory of pictures over sounds by comparing recognition performance in same day testing and delayed testing. They used the exact same stimulus set as Cohen et al. (2009). Gloede and Gregg (2019) presented pictures or sounds in a study phase and participants were tested on their yes–no recognition memory of targets (items presented during study), exemplar foils (new exemplars from the same categories as studied stimuli), and novel foils (new stimuli categorically distinct from any studied stimuli). This separation of new items into exemplar foils and novel foils enabled Gloede and Gregg (2019) to determine the degree to which retrieval of gist and verbatim information contributed to higher recognition memory for pictures compared with sounds. To discriminate an exemplar foil from a studied item, one would need to retrieve detailed or verbatim-based information, whereas to discriminate a novel foil from a studied item, retrieving gist-based information would be sufficient for correct recognition. Moreover, to correctly recognise a studied item, one would not only need to retrieve gist-based information but also some verbatim-based information.

The results of the second experiment by Gloede and Gregg (2019) in which the study and test phases were on the same day, showed that percent correct on the visual memory test was high and uniform across all probe conditions. Moreover, Gloede and Gregg (2019) replicated the findings by Cohen et al. (2009, 2011) by showing that recognition accuracy was higher for pictures compared with sounds. However, performance in the auditory memory task was not uniform. Gloede and Gregg (2019) suggested the lower recognition accuracy for exemplar foils indicated that auditory memory representations are less perceptually detailed or not of as high fidelity as visual memory representations.

Gloede and Gregg (2019) concluded that the recognition advantage for pictures on the same-day test was due to LTM representations of auditory stimuli being coarser and gist-based, whereas visual representations are more perceptually detailed. The results and interpretation by Gloede and Gregg provide valuable insight into the mechanism for the recognition advantage for pictures over sounds. However, there are reasons to pursue an extension and modification of their study design.

## Purpose and rationale of this study

In this study, we aimed to test fuzzy-trace and the conceptual-distinctiveness theories, which can potentially explain the superior recognition memory observed for pictures in comparison with sounds. Both theories can explain why pictures are remembered better than sounds, the difference being that fuzzy-trace theory focuses on the nature of representations created during memory encoding, whereas conceptual-distinctiveness theory emphasises how visual information supports the semantic processing of the item and the resulting conceptual representation. According to fuzzy-trace theory, we suggest that pictures would typically be better remembered than sounds due to greater retention of verbatim information. Whereas, according to a conceptual distinctiveness account, we suggest that pictures would be better remembered than sounds because there is deeper level of processing (Craik & Lockhart, 1972) induced by identifying a picture than a sound. For example, Hockley (2008; Hockley & Bancroft, 2011), using an associative recognition test, showed that pairs of pictures were recognised better than pairs of words and theorised that picture pairs received more semantic processing than word pairs, which would follow with a conceptual-distinctiveness theory.

Further support for a conceptual distinctiveness account comes from Konkle et al. (2010a). The researchers found that as the number of exemplars within a category increased, the decline in recognition memory performance for pictures of objects was not mediated by the degree of perceptual distinctiveness (i.e., shape, colour) but rather conceptual distinctiveness (i.e., kind of object). In their second experiment, they obtained ratings of conceptual and perceptual distinctiveness for exemplars within categories. Konkle et al. (2010a) found that categories composed of conceptually distinct items were relatively spared from interference with increasing numbers of exemplars in memory. Konkle et al. (2010a) concluded that conceptual distinctiveness played a prominent role in supporting detailed representations in visual LTM.

Of course, there are other theories than the conceptual-distinctiveness theory. The alternative theories being dual-coding and the physical-distinctiveness theories for which support has also come from the investigation of recognition memory of pictures compared with words. According to Paivio’s (1991) dual-coding theory, pictures are more likely than words to be represented in both a verbal and an imaginal form. In contrast, according to the physical-distinctiveness theory, it is the physical features of pictures that make it distinctive in memory from other stimuli. In support of the physical distinctiveness account of the PSE, Ensor, Surprenant, and Neath (2019) showed that the PSE could be reduced or eliminated when words were made physically distinctive by varying font style, font size, colour, and capitalization, and the PSE could even be reversed when the physical distinctiveness of pictures was reduced.

What adds further complexity to the understanding of the mechanisms that could account for the higher recognition memory of pictures compared with sounds, is that a combination of theories could contribute to our understanding of the PSE.^1^ For example, pictures may be more prone to be dual coded compared with sounds, and as a result, pictures would be more conceptually and perceptually distinctive than sounds as dual coding increases both meaning and perceptual details of pictures. Consequently, there would be stronger verbatim and gist representation in memory for pictures compared with sounds. For example, discriminating between a basketball and a soccer ball relies on both perceptual and conceptual information, such as differences in colour and association with different sports. Thus, based on fuzzy-trace theory, conceptual-distinctiveness theory, and dual-coding theory, one could make similar predictions on the general distinctions between verbatim and gist information, but the specific details of the distinctions would differ.

## Aims and predictions of this study

Experiments 1A and 1B were designed to test fuzzy-trace theory’s predictions by extending the research study of Gloede and Gregg (2019). In Experiment 1A, pictures and sounds were presented in mixed rather than separate lists. In Experiment 1B, we determined whether their findings for their same day yes–no recognition test could generalise to a two-alternative forced-choice (2AFC) recognition test. Research has shown that the 2AFC test provides a more direct test of recognition based on gist versus perceptual details (Ahmad et al., 2017; Andermane & Bowers, 2015; Brady et al., 2008; Konkle et al., 2010b). There are two test conditions in the 2AFC test that follows the study phase.

In the novel test condition, a new item such as a picture from a category that was not present during the study phase is presented with a target item. One could discriminate the target from the new item based on memory for category- or gist-based information. In contrast, in the exemplar test condition, a target is paired with an exemplar foil that was not presented during the study phase but is an exemplar from the same category as the target. Discriminating between these alternatives must be based on memory for perceptual details as category information is not informative. The sensitivity of this 2AFC test procedure to distinguish differences in retrieval of gist and verbatim information in the novel and exemplar test conditions has been shown by Andermane and Bowers (2015), Konkle et al. (2010b), and Ahmad et al. (2017). In the study by Konkle et al. (2010b), participants viewed 2,912 images from 128 different scene categories, with 1, 4, 16, or 64 exemplars presented per category. Recognition memory was highest in the novel test condition (i.e., 96%) and, as expected, recognition memory decreased with an increase in the number of exemplars during the study phase (i.e., 76% for 64 exemplars).

Furthermore, the retrieval of gist and verbatim information during the recognition test phase has also been shown to be dependent on encoding time. Ahmad et al. (2017) compared recognition in the 2AFC test for scenes presented for 4 s to scenes presented for 1 s during the study phase. Less verbatim-based information (i.e., surface, or perceptual details) and more gist-based information (i.e., meaning) of a scene was retrieved from LTM after a short compared with a long study presentation consistent with the encoding assumptions of fuzzy-trace theory.

We predicted recognition accuracy would be significantly lower in the exemplar test condition for sounds compared with pictures, showing that more verbatim information is encoded and retrieved in correct recognition of pictures compared with sounds. We also included Tulving’s (1985) remember-know response procedure to determine if the recognition advantage for pictures over sounds is also associated with greater recollection of the pictures. We predicted there would be significantly more remember responses shown for pictures than sounds.

Experiments 2A and 2B tested predictions according to a combination of dual-coding, conceptual distinctiveness, and fuzzy-trace theories. We investigated whether the presentation of verbal labels for sounds during encoding and testing would eliminate the superior recognition memory for pictures. We predicted such manipulations would reduce the recognition advantage for pictures, because the presentation of verbal labels would increase the semantic processing of sounds.

According to Paivio’s (1991) dual-coding theory, pictures are more likely than words to be represented in both a verbal and an imaginal form. A concrete explanation for the similarity and differences between recognition memory for pictures, words, and sounds has yet to be established. Ensor, Bancroft, and Hockley (2019) were not able to find a recognition memory advantage for environmental sounds compared with words. In their experiments, sounds and words were presented during the study followed by a test phase where participants provided old and new judgements for studied and new words and sounds. They found that there was similar recognition accuracy between sounds and words. Furthermore, they found that there was a similar proportion of remember responses for sounds and words based on Tulving’s (1985) remember/know response procedure.

Hamilton and Geraci (2006) directly related the PSE to the processing and identification of a picture’s diagnostic features. As an example of how visual information can contribute to conceptual distinctiveness for an item, Hamilton and Geraci (2006) suggested to consider a picture of an owl. Its wings are diagnostic of its identity as a bird, but they do not inform the perceiver of the type of bird. The owl’s large eyes, however, distinguish an owl from other birds; thus, its large eyes are more diagnostic than its wings. In the case of pictures, perceivers need to actively determine which features are most diagnostic, however this process would be more difficult with sounds, as sounds are presented in temporal as opposed to spatial distribution as in the case of pictures. As Ensor, Bancroft, and Hockley (2019) noted, perceivers cannot devote more time to some features of sounds over other features because sounds are distributed temporally rather than spatially.

Importantly, in the above studies, the sounds largely consisted of diagnostic features presented in isolation. That is, physical and perceptual features for sounds were presented out of context. Thus, Ensor, Bancroft, and Hockley (2019) proposed a hybrid account of the PSE in which the PSE in free recall stems from the greater dual coding of pictures and sounds compared with words, whereas the PSE in recognition is due to conceptual distinctiveness arising from the nature of the processing of pictures. There is no advantage for sounds over words in tests of recognition because of the absence of conceptual distinctiveness. However, as we will show in this study, conceptual distinctiveness for sounds can be increased by presenting them with verbal labels, which act to provide context to the sounds to be encoded and retrieved from LTM.

Finally for Experiments 3A and 3B, we wanted to examine if the perceptual or conceptual distinctiveness of pictures could account for higher recognition memory of pictures compared with sounds by determining whether the similarity of pictures had an influence on the recognition advantage for pictures. We examined the relation between ratings of stimulus similarity and recognition accuracy. We had participants provide the basis of their similarity judgement in terms of semantic features (i.e., category, function) and perceptual features (i.e., colour, shape, pattern, pitch, loudness). The reasoning was that test pictures could be rated as less similar to each other compared with sounds and this could account for higher recognition memory for pictures. Thus, it was either the conceptual or perceptual distinctiveness of pictures that lead to higher recognition memory for pictures compared with sounds. Furthermore, if pictures are less similar with each other compared to sounds, the difference in similarity would lead to more verbatim details to be encoded for pictures than sounds leading to higher memorability for pictures compared with sounds. We predicted similarity would be lower for pictures than sounds, which would account for the higher recognition memory for pictures compared with sounds. Distinctiveness or reduced similarity within a stimulus group has shown to lead to higher recognition memory for the individual stimuli as in pictures of objects and scenes (Brady et al., 2008; Konkle et al., 2010a).

## Experiment 1A

In Experiment 1A, we examined whether there would be better recognition memory for pictures compared with sounds when pictures and sounds were presented in separate trials in a mixed list during the study and test phases. As noted earlier, Cohen et al. (2009, 2011), Gloede et al. (2017), and Gloede and Gregg (2019) presented pictures and sounds separately in two different study and test blocks. Moreover, in both the study and yes–no recognition test phases of this experiment, the pictures were categorically distinct from the sounds. Participants also provided a confidence judgement for each recognition decision. We predicted lower recognition memory for sounds compared with pictures as shown by poorer discrimination of targets, exemplar foils, and novel foils. This prediction was based on our assumption shared with Gloede and Gregg (2019) that less gist and verbatim information are encoded at study and retrieved at test for sounds than for pictures. Based on the findings of Ahmad et al. (2017), we also predicted lower confidence for gist-based than verbatim-based decisions, because retrieval of perceptual details would elicit more confidence in the accuracy of memory.

## Methods

The data from our experiments, R code, and Stimulus set adapted from Gloede and Gregg (2019) have been made publicly available on the Open Science Framework: https://osf.io/fz6tk/.

### Participants

All participants in each experiment were recruited through the Department of Psychology’s research participation programme at Wilfrid Laurier University. They received course credit for participation. Participants only participated in one of the experiments. Twenty-two undergraduate students (*Mean age* = 19.6, *SD* = 1.30; 12 females) participated in Experiment 1. All institutional ethics review board procedures were followed, and consent was received from all participants.

The sample size for this experiment and subsequent experiments in this study were comparable to the sample sizes in the studies of Cohen et al. (2009) and Gloede and Gregg (2019) who used the same stimuli. It should be noted that in Experiment 2 of Gloede and Gregg (2019) study, there were large values of partial effect size (i.e., partial eta squared) reported, with a sample size of 20 participants and implementation of a two-way repeated measures (2[ Type: Picture, Sound] × 3[Memory Probe: Target, Exemplar foil, Novel foil]) design. For their significant effect of Memory type (i.e., stimulus type), Gloede and Gregg (2019) had a partial eta squared value of .74 and for Memory Probe (i.e., test probe), a partial eta squared value of .27. Finally, for the interaction they reported a partial eta squared value of .15.^2^

We conducted a post hoc sample size power analysis in the following steps using G*power software (Kang, 2021; Serdar et al., 2021).^3^ Our post hoc analyses showed that our sample sizes for each experiment were adequate. Bayesian analysis was implemented to assess any null effects in the case of the interaction.

### Apparatus and stimuli

All experiments were conducted online due to COVID-19 restrictions. Participants used their own laptop or desktop computer to run the online experiment. Gorilla Experiment Builder (gorilla.sc) was used to code or create the experiment and control stimulus presentation and response recording. Using a desktop or laptop computer, the Gorilla platform has shown to be effective in running sensitive reaction time experiments and has replicated well-known paradigms such as the flanker test (Anwyl-Irvine et al., 2020, 2021). The stimulus set was adapted from Gloede and Gregg (2019) and consisted of 96 pictures of common categorically distinct objects (e.g., picture of dog barking, apple) and 96 common categorically distinct environmental sounds, each of which corresponded to one of the pictures (e.g., the sound of dog barking corresponded to the picture of a dog barking). To note, in their stimulus set, there were two pictures and two sounds associated with an autocar scene and a bowling alley scene, portraying scenes rather than individual objects. All sounds were 5 s in duration, digitised to a sampling rate of 44.1 kHz, matched for root mean square (RMS) amplitude, filtered for noise, and off-ramped to avoid abrupt transients (Gloede & Gregg, 2019).

A brief explanation of the stimulus set used in Gloede and Gregg (2019) research study should be provided together with an explanation of the necessity to add additional sounds and pictures for this study. First, the sounds were 5 s in duration to match with picture presentation of 5 s, and as a result all sounds such as a “dog barking” were repeated to fill the 5 s presentation rate. One limitation in the presentation of sounds in our research study was that the degree of repetition of the sound would vary by the length of the sound. For example, sounds of short duration such as a “dog barking” were repeated more than sounds of long duration to fill in the 5 s presentation rate. Second, some of the sounds presented were dynamic in the representation of pictures of static objects. For example, for the sound equivalent of a picture of a rocking chair, the sound of a rocking chair was presented. Finally, in the study of Gloede and Gregg (2019), there were 64 pictures or 64 sounds presented during the study phase. The test phase consisted of half of the study phase sounds, 16 novel sound objects that were categorically distinct from the sounds used in the study phase, and 16 exemplar sound objects that were different exemplars from the same category as sounds presented during the study phase. As a result, 96 pictures or 96 sounds were presented in the experiment.

However, in the study by Gloede and Gregg (2019), for only 16 pictures and 16 sounds, there were exemplar foils, and those exemplar foils were always presented during the test phase for each participant. In contrast, for the other 48 pictures and 48 sounds, no exemplar foils were presented at test. Moreover, there was no counterbalancing of study and test items implemented. For example, the novel pictures and sounds were always presented as novel pictures and sounds to each participant.

We added an additional 10 picture and 10 sound exemplar foils to the stimulus set, along with an additional 10 novel pictures and 10 novel sounds, due to changes in the procedures of Experiments 1A and 1B. As a result, within the stimulus set being used, there were 116 pictures and 116 sounds. This was necessary to counterbalance the pictures and sounds as targets and exemplars across participants, while avoiding the presentation of similar pictures with sounds in the same study and test lists. The novel items were always counterbalanced with targets.

To achieve this aim, we completed two steps. We collected additional pictures from the Google image database and sounds from the Free sound website (freesound.org) that we agreed on being representative of category and similar to the Gloede and Gregg (2019) stimulus set of pictures or sounds. To confirm the added exemplars to the stimulus set were representative of category and similar to the exemplar of similar category, 20 undergraduate students viewed the added pictures and sounds (i.e., exemplar) paired with similar picture or sound from Gloede and Gregg (2019) stimulus set. On each trial, participants would be presented with two sounds or two pictures, one of the sounds and one of the pictures on each trial would be from Gloede and Gregg (2019) stimulus set. Participants were to indicate on a scale of 1 to 4, which of the two pictures or sounds was most similar to picture or sound in stimulus set and representative of the category. We selected the pictures and sounds with the highest mean ratings of similarity. For post hoc analysis, we provide the mean similarity analysis from Experiment 3B in terms of comparison of similarity between new set of pictures and sounds with that of Gloede and Gregg (2019) stimulus set.^4^

Four sets of pictures and sounds were created for counterbalancing. The sounds and pictures presented as targets and exemplars were counterbalanced across participants. The novel items were also counterbalanced with targets. For the study phase, 26 pictures of categorically distinct pictures of objects and scenes and 26 categorically distinct sounds were constructed. For the test phase, as there were targets, exemplar foils, and novel items presented, 13 pictures and 13 sounds were presented as targets with an additional 13 pictures and 13 sounds presented as exemplar foils for pictures and sounds. A further 13 pictures and 13 sounds were presented as novel pictures and sounds. As a result, a total of 78 stimulus items (39 pictures and 39 sounds) were presented during the test phase.

### Procedure

Participants attended a virtual meeting with an experimenter on the video conferencing platform called Zoom. This was an important part of the procedure, to make sure that participants could see instructions, view pictures on their computer screen, and hear the sounds.^5^ The consent form and study instructions were read and explained by the experimenter to the participant. A brief practice phase was completed via Gorilla.sc where participants were asked to describe pictures and sounds presented to them that were not presented during the experiment, to ensure they understood instructions and could see or hear the stimuli presented. At the end of the meeting, participants were given a web link to complete the experiment on the Gorilla.sc platform. The experimenter confirmed with the participant that they were alone in a quiet room and verified that they started the experiment once the participant was provided the web link. The experiment was launched by the web link, which was compatible with any browser and operating system.

At the beginning of the study phase, an audio recording of the experimenter reading the study instructions was presented. Participants were told they would be presented with pictures and sounds, after which there would be a recognition test for the pictures and sounds. Shortly after the study instructions were shown to the participant and once the participant pressed the space bar, the study phase began. During the study phase, pictures and sounds were presented in a randomised order, with a different random order for each participant. For each trial, a fixation cross would be presented for 250 ms followed by a picture or sound presented for 5 s. In total, 26 pictures and 26 sounds were presented during the study phase. After the study phase, instructions were provided on the screen indicating which keys to press to indicate the old and new responses (1-*Old*, 2-*New*), followed by using the mouse for their confidence judgement (certain no, probably no, guess no, guess yes, probably yes, certain yes). The instructions also indicated to select one of the confidence ratings: “certain no,” “probably no,” or “guess no” only when they had responded “New” and “certain yes,” “probably yes,” or “guess” when they had responded “Old” to a test stimulus.

**Figure 1.**
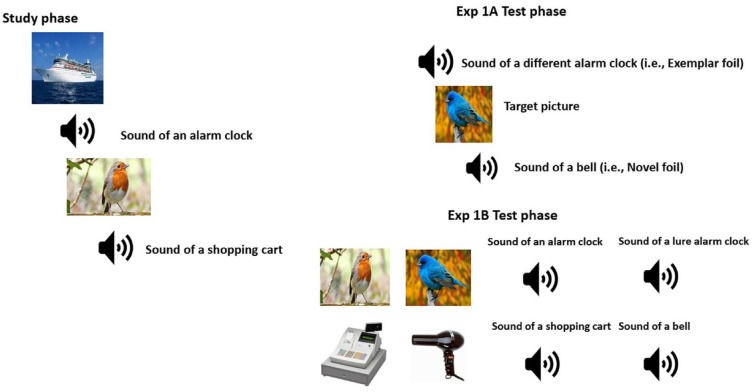
Displayed is the study phase with a sample of list of pictures and sounds. The study phase was similar for Experiments 1A and 1B. Displayed are sample test items presented in the yes–no recognition test of Experiment 1A (exemplar foil, novel foil, target) and the exemplar and novel test conditions presented in the 2AFC recognition test of Experiment 1B. Figures of fixation boxes with interstimulus interval (ISI) of 250 ms during the study phase and ISI of 125 ms during the test phase, are not included.

For each test trial, a fixation cross was presented for 125 ms, after which a picture or sound was presented for 5 s. Immediately after the picture or sound had been presented, test instructions were displayed prompting participants to indicate by pressing 1 or 2 on their keyboard whether the picture or sound was a studied item. After providing their old or new judgements, test instructions were displayed indicating to participants to provide their confidence judgement. In total, 39 pictures and 39 sounds were presented in a different random order for each participant during the test phase. Figure 1 displays examples of the stimuli and test conditions for Experiment 1A.

## Results

### Accuracy

A 2 (stimulus type: picture vs sounds) × 3 (test probe: targets, exemplar foil, novel foil) repeated analysis of variance (ANOVA) was conducted on proportion of correct responses. Mean proportions of correct responses to test probes in Yes/No recognition memory test are shown in Table 1. Accuracy is defined by correct rejection of exemplar foils and hit rate for targets. For example, higher accuracy in recognition memory for pictures would be evidenced by a higher correct rejection rate of exemplar foil pictures and higher hit rate for target pictures compared with sounds.

**Table 1.** Mean proportions of correct responses for targets, exemplar foils, and novel foils for pictures and sounds in Experiments 1A, 2A, and 2B.

	Target	Exemplar foil	Novel foil
Experiment 1A			
Pictures	0.91 (0.09)	0.72 (0.16)	0.98 (0.04)
Sounds	0.79 (0.17)	0.58 (0.17)	0.87 (0.16)
Experiment 2A			
Pictures	0.90 (0.09)	0.70 (0.13)	0.97 (0.06)
Sounds	0.72 (0.16)	0.59 (0.18)	0.90 (0.09)
Experiment 2B			
Pictures	0.83 (0.14)	0.68 (0.16)	0.96 (0.04)
Sounds	0.84 (0.12)	0.38 (0.21)	0.93 (0.09)

The standard errors of the means are shown in parentheses.

There was a significant main effect of stimulus type, *F*(1, 21) = 52.56, *MSE* = 0.010, *p* = .001, *η*^2^_*p*_ = .715. Higher accuracy was shown for pictures (*M* = 0.87, SE = 0.014) compared with sounds (*M* = 0.74, *SE* = 0.023). There was also a significant main effect of test probe, *F*(2, 42) = 51.7, *MSE* = 0.017, *p* < .001, *η*^2^_*p*_ = .711. Higher accuracy was shown for novel foils (*M* = 0.92, *SE* = 0.019) compared with targets (*M* = 0.85, *SE* = 0.021), with lowest accuracy for exemplar foils (*M* = 0.65, *SE* = 0.029). Pair-wise comparisons showed there to be higher accuracy for novel foils compared with targets (*d* = 0.0734, *p* = .024) and higher accuracy for targets compared with exemplar foils (*d* = 0.198, *p* < .001). There was no significant interaction between stimulus type and test probe, *F*(2, 42) = 0.208, *MSE* = 0.020, *p* = .813, *η*^2^_*p*_ = .010.

### Bayesian analysis

We used Bayesian analysis implemented in JASP (based on tutorial of Bayesian analyses by Masson [2011], as detailed by Dienes, 2014; Wagenmakers, 2007; Wagenmakers et al., 2018) to assess the power of the interaction for which effect sizes led to concern that a much larger sample size would have produced a significant effect. We used the Bayes factor (BF), which is a Bayesian model comparison technique (Kass & Raftery, 1995; Morey et al., 2016). The BF is a ratio between the evidence for one model given the data and the evidence for another model given the data. If the BF for the alternative hypothesis (H_1_) model relative to the null hypothesis (H_0_) model is X, then H_1_ is X times more likely given the data than H_o_.

For Experiment 1A, to evaluate the evidence for the interaction on its own, we compared the BF_10_ of the model with the interaction against the BF_10_ of the model with only the two main effects (i.e., everything except the interaction) as shown in the study by Wagenmakers et al. (2018). We found the evidence for the interaction was 7.126 × 10^10^ (interaction)/4.213 × 10^11^ (main effects) = 0.172, which is less than 1. We used guidelines established by Wetzels et al. (2011) for determining weak, moderate, and strong evidence for alternative hypothesis based on BF. According to the guidelines, a BF of 1 indicates no evidence in support for the alternative hypothesis, whereas a BF between 1 and 3 indicates anecdotal evidence for alternative hypothesis and between 3 and 10 indicates substantial evidence for alternative hypothesis. Thus, there was no evidence to support the interaction as one would expect given the traditional repeated measures.

### D prime

The *d′* and criterion estimates (*c*) for targets, exemplar foils, and novel foils for pictures and sounds are shown in Table 2.

**Table 2.** The *d*′ and criterion estimates for targets, exemplar foils, and novel foils for pictures and sounds in Experiments 1A, 2A, and 2B.

	*d*′ Target– Novel foil	*d*′ Target–Exemplar foil	Criterion Target–Novel foil	Criterion Target–Exemplar foil
Experiment 1A				
Pictures	3.04 (0.46)	2.05 (0.62)	0.15 (0.21)	−0.35 (0.35)
Sounds	2.15 (0.91)	1.16 (0.76)	0.15 (0.40)	−0.34 (0.41)
Experiment 2A				
Pictures	2.94 (0.53)	1.88 (0.69)	0.14 (0.23)	−0.38 (0.23)
Sounds	2.00 (0.55)	0.93 (0.67)	0.33 (0.38)	−0.20 (0.41)
Experiment 2B				
Pictures	2.69 (0.63)	1.60 (0.84)	0.26 (0.28)	−0.28 (0.31)
Sounds	2.59 (0.79)	0.76 (0.74)	0.18 (0.26)	−0.73 (0.44)

The standard errors of the means are shown in parentheses.

Two *d*′ scores were calculated. One as a measure of discrimination of targets from novel foils (i.e., target hit rate vs novel false alarm rate) and the other for discrimination of targets from exemplar foils (i.e., target hit rate vs exemplar foil false alarm rate). Thus, *d*′ (T, NF) reflects the difference in distributions between old responses to targets and old responses to novel foils. In contrast, *d*′ (T, EF) reflects the difference in distributions between old responses to targets and old responses to exemplar foils. A 2 (Stimulus Type: picture vs sounds) × 2 (*d*′ Type: target–novel foil vs target–exemplar foil) repeated measures ANOVA was conducted on *d*′ estimates. There was a significant main effect of stimulus type, *F*(1, 21) = 34.5, *MSE* = 0.508, *p* < .001, *η*^2^_*p*_ = .622. Higher *d*′ score was shown for pictures (*M* = 2.54, SE = 0.105) compared with sounds (*M* = 1.66, *S.E* = 0.162). There was also a significant main effect of *d*′ type, *F*(1, 21) = 148.12, *MSE* = 0.146, *p* = .000, *η*^2^_*p*_ = .877. Higher accuracy was shown in the target–novel foil (*M* = 2.60, *SE* = 0.118) than in the target–exemplar foil (*M* = 1.60, *SE* = 0.122) test condition. There was no significant interaction between stimulus type and *d*′ type, *F*(1, 21) = 0.001, *MSE* = 0.236, *p* = .973, *η*^2^_*p*_ = .000.

#### Bayesian analysis

We found the evidence for the interaction was 9.861 × 10^9^ (interaction)/3.240 × 10^10^ (main effects) = 0.30. Thus, there was no evidence in support of the interaction.

### Criterion

Estimates of criterion placement are also shown in Table 2. A 2 (stimulus type: picture vs sounds) × 2 (criterion type: target–foil vs target–exemplar foil) repeated measures ANOVA showed there was no significant main effect of stimulus type, *F*(1, 21) = 0.003, *MSE* = 0.193, *p* = .954, *η*^2^_*p*_ = .000. Mean criterion was similar for pictures (*M* = –0.100, *SE* = 0.056) and sounds *(M* = –0.095, *SE* = 0.077). However, there was a significant main effect of criterion type, *F*(1, 21) = 149.12, *MSE* = 0.036, *p* < .001, *η*^2^_*p*_ = .877. Mean criterion was more positive (i.e., conservative) for target–novel foils (*M* = 0.151, *SE* = 0.047) than target–exemplar foils *(M* = –0.346, *SE* = 0.057). There was no significant interaction of stimulus type and criterion type, *F*(1, 21) = 0.001, *MSE* = 0.059, *p* = .873, *η*^2^_*p*_ = .000.

### Confidence

Mean confidence scores for correct responses to each test probe are shown in Table 3.

**Table 3.** Mean confidence for correct responses to targets, exemplar foils, and foils for pictures and sounds in Experiments 1A, 2A, and 2B.

	Target	Exemplar foil	Novel foil
Experiment 1A			
Pictures	2.83 (0.16)	2.49 (0.69)	2.80 (0.23)
Sounds	2.57 (0.38)	2.26 (0.60)	2.41 (0.51)
Experiment 2A			
Pictures	2.88 (0.17)	2.56 (0.41)	2.66 (0.33)
Sounds	2.69 (0.24)	2.30 (0.44)	2.47 (0.39)
Experiment 2B			
Pictures	2.81 (0.13)	2.37 (0.48)	2.44 (0.57)
Sounds	2.77 (0.18)	2.30 (0.54)	(0.38)

Four participants responded with inconsistent confidence ratings for correct and incorrect responses to the test probe (e.g., giving a confidence judgement of “probably new” after giving an “old” recognition response). Specifically, the four participants did not show any correct “certain no,” “guess no,” and “probably no” responses for exemplars and novel items. However, these same four participants showed correct “certain yes,” “guess,” and “probably yes” responses to targets. As a result, the confidence data from these participants were not included in the analysis. The confidence judgements were numerically coded as 3 (*certain old or new*), 2 (*probably old or new*), and 1 (*guess old or new*). For statistical analysis, we focused on mean confidence related to correct responses to targets, exemplar foils, and novel foils as confidence judgements for incorrect responses are based on fewer responses and are thus less reliable. A 2 (stimulus type: picture vs sounds) × 3 (test probe: target vs exemplar foil vs novel foil) repeated measures ANOVA revealed a significant main effect of stimulus type, *F*(1, 17) = 14.1, *MSE* = 0.214, *p* = .002, *η*^2^_*p*_ = .453. Higher confidence was shown for pictures (*M* = 2.75, *SE* = 0.05) compared with sounds (*M* = 2.41, *SE* = 0.10). There was also a significant main effect of test probe, *F*(2, 34) = 8.07, *MSE* = 0.078, *p* = .001, *η*^2^_*p*_ = .322. Pair-wise comparisons showed higher confidence in correct recognition for targets (*M* = 2.70, *SE* = 0.05) compared with exemplar foils (*M* = 2.44, *SE* = 0.10), *p* = .003, yet similar confidence for novel foils (*M* = 2.60, *SE* = 0.08) and exemplar foils (*M* = 2.44, *SE* = 0.10), *p* = .245. There was no significant interaction between stimulus type and test probe, *F*(2, 34) = 1.069, *MSE* = 0.048, *p* = 0.355, *η*^2^_*p*_ = .059.

## Discussion

We showed, using a yes–no recognition test with both pictures and sounds presented during the study phase, that participants had higher recognition memory for pictures compared with sounds. Specifically, participants showed both a higher hit rate for targets and lower false alarm rates for novel foils and exemplar foils for pictures compared with sounds, a pattern referred to as the mirror effect (Glanzer & Adams, 1985). For both pictures and sounds, participants showed lowest recognition accuracy for exemplar foils. Our results are similar to that of Gloede and Gregg (2019) in that we found higher recognition memory for pictures compared with sounds. Interestingly, we found a higher hit rate for picture targets and higher correct rejection rate for novel foils and exemplar foils compared with sound targets, novel foils, and exemplar foils. In contrast, Gloede and Gregg (2019) only reported a higher correct rejection rate for picture exemplar foils compared with sound exemplar foils. Specifically, they found in their second experiment, in which testing was on the same day, a significant interaction between stimulus type and test probe (i.e., *p* = .049). Gloede and Gregg (2019) interpreted this interaction to indicate that recognition was higher for pictures than sounds due to greater retrieval of perceptual details. In contrast, we did not find an interaction between stimulus type and test probe, showing that higher recognition of pictures compared with sounds is due to both retrieval of a higher quantity of gist and verbatim information.

To extend the findings by Gloede and Gregg (2019), we showed that there was also a higher *d*′ estimate shown for target–novel foil discrimination compared with target–exemplar foil discrimination, indicating that the retrieved gist information was more informative than the retrieved verbatim information. Mean criterion estimates were more positive or conservative for pictures compared with sounds. However, our estimates of *d*′ and conclusions based on them must be interpreted with some caution. A better measure would be *da*, which requires calculation of receiver operating characteristics (ROCs) that we were not able to perform as we did not have enough stimuli per stimulus category. As noted by Brady et al. (2023) and Dougal and Rotello (2007), *d*′ is accurate only if all items are encoded equally well. The confidence rating results showed that there was higher confidence in recognition of pictures compared with sounds. Yet, there was similar confidence in correct rejections of exemplar and novel foils with highest mean confidence for targets.

## Experiment 1B

In Experiment 1B, we wanted to see if the lower recognition performance for sounds compared with pictures as shown in the yes–no recognition test would generalise to a 2AFC recognition test. The 2AFC test provides a more direct test of recognition based on gist versus perceptual details (Ahmad et al., 2019; Konkle et al., 2010a). We predicted lower recognition for sounds compared with pictures in both the exemplar and the novel test conditions. This prediction was based on assumption that lower recognition memory for sounds compared with pictures is due to reduced retrieval of both gist and verbatim information. Moreover, we implemented a remember/know/guess judgement to access the relative use of recollection and familiarity in the recognition of pictures and sounds in the 2AFC test. These remember-know-guess (RKG) instructions were adapted from the study by Kuhbandner (2020) but modified to take account of the stimulus change. We adopted a dual process view of measurement of remember and know responses that follows with Yonelinas and Jacoby (1995). Remembering and knowing reflect qualitative different traces (Dual process theories) rather than reflecting decision processes in memory (single process theories) (Gardiner et al., 1998). Remembering reflects a consciously controlled form of memory, characterised by recollection (i.e., retrieving from memory contextual details of the picture or sound). In contrast, knowing reflects an automatic form of memory, characterised by familiarity (i.e., not remembering any contextual details, but experiencing a phenomenal feeling for knowing). In contrast, guess responses are based on an absence of relevant information (i.e., no phenomenal experience of recollection or familiarity) (Gardiner & Richardson-Klavehn, 2000; Kuhbandner, 2020).

## Methods

### Participants

Twenty-two (*Mean age* = 19.3, *SD* = 1.46; 14 females) undergraduate students participated for course credit.

### Materials

The stimuli and the counterbalancing of the study and test lists were the same as in Experiment 1A. For the 2AFC test phase, the exemplar test was constructed by the pairing of a study item with a new item from the same category. The novel test was constructed by the pairing of a study item with an item from a novel category that was not represented in the study stimulus set.

### Procedure

The procedure was the same as in Experiment 1A, except for the following changes. Immediately after the study phase, instructions were provided on the screen indicating which keys to press to indicate the choice of studied item (1—for *first choice* or 2—for *second choice*), followed by pressing a key for RKG judgement (I—*Remember*, O—*know*, P—*Guess*). In the test phase, half of the trials were target-exemplar foil trials whereas the other half were target-novel foil trials. During the test trial, a fixation cross would be presented for 125 ms, after which a picture or sound would be presented for 5 s followed by fixation for 125 ms and then the second picture or sound would be presented for 5 s. Participants would then be prompted to indicate which picture or sound was a studied item, after which participants were to provide their RKG judgement. During the test phase, there were 26 picture test trials and 26 sound test trials. For each participant, both the study and test trials were presented in a randomised order. Figure 1 displays examples of the stimuli and test conditions for Experiment 1B. Accuracy, R, K, and G responses were recorded.

## Results

### Accuracy

Proportion of correct responses to target in each test condition by stimulus type are shown in Table 4.

**Table 4.** Mean proportions of correct responses and mean proportions of remember, know, and guess (RKG) judgements for correct responses in the exemplar and novel test conditions for pictures and sounds for Experiment 1B.

Target correct	Remember	Know	Guess	Total proportion correct for target
Picture_Exemplar	0.55 (0.28)	0.23 (0.19)	0.08 (0.11)	0.86 (0.35)
Sound_Exemplar	0.41 (0.18)	0.28 (0.16)	0.11 (0.08)	0.80 (0.40)
Picture_Novel	0.72 (0.21)	0.16 (0.18)	0.07 (0.10)	0.96 (0.20)
Sound_Novel	0.43 (0.17)	0.25 (0.15)	0.17 (0.13)	0.86 (0.34)

The standard deviations of the means are shown in parentheses.

A 2 (stimulus type: picture vs sounds) × 2 (test condition: exemplar vs novel) repeated measures ANOVA was conducted on the proportion of correct responses. There was a significant main effect of stimulus type, *F*(1, 23) = 11.40, *MSE* = 0.011, *p* = .003, *η*^2^_*p*_ = .331. Higher accuracy was shown for pictures (*M* = 0.91, *SE* = 0.014) compared with sounds (*M* = 0.83, *SE* = 0.021). There was also a significant main effect of test condition, *F*(1, 23) = 11.87, *MSE* = 0.012, *p* = .002, *η*^2^_*p*_ = .340. Higher accuracy was shown in the novel (*M* = 0.91, *SE* = 0.012) than in the exemplar (*M* = 0.83, *SE* = 0.023) test condition. There was no significant interaction between stimulus type and test condition, *F*(1, 23) = 1.816, *MSE* = 0.005, *p* = .191, *η*^2^_*p*_ = .073.

### Bayesian analysis

For Experiment 1B, we found the evidence for the interaction was 165.40 (interaction)/279.74 (main effects) = 0.154. Thus, there was no evidence in support of the interaction.

### RKG judgement

Proportions of remember (R), know (K), and guess (G) judgements for correct responses are provided in Table 4. As RKG responses are mutually exclusive (i.e., dependent on each other), we could not enter these responses into a single ANOVA as would violate the assumption of independence, as noted by Haaf et al. (2021) and Yonelinas and Jacoby (1995). As a result, we conducted three separate ANOVAs to analyse R, K, and G responses separately.

A 2 (stimulus type: pictures vs sounds) × Probe (exemplar foil, novel foil) repeated measures ANOVA was conducted on proportion of R responses. There was a significant main effect of stimulus type, *F*(1, 23) = 35.8, *MSE* = 0.030, *p* < .001, *η*^2^_*p*_ = .609. There was a higher proportion of remember responses for pictures (*M* = 0.636, *SE* = 0.032) than sounds (*M* = 0.425, *SE* = 0.025). There was no significant main effect of test probe, *F*(1, 23) = 3.617, *MSE* = 0.070, *p* = .070, *η*^2^_*p*_ = .136. There was no significant interaction between stimulus type and test probe, *F*(1, 23) = 3.81, *MSE* = 0.034, *p* = .063, *η*^2^_*p*_ = .142.

A 2 (stimulus type: pictures vs sounds) × Probe (exemplar foil, novel foil) repeated measures ANOVA was conducted on proportion of K responses. There was a strong trend for a main effect of stimulus type, *F*(1, 23) = 3.99, *MSE* = 0.029, *p* = .058, *η*^2^_*p*_ = .148. The proportion of know responses was lower for pictures (*M* = 0.197, *SE* = 0.032) than sounds (*M* = 0.266, *SE* = 0.028). There was no significant main effect of test probe, *F*(1, 23) = 3.83, *MSE* = 0.014, *p* = .062, *η*^2^_*p*_ = .143. There was no significant interaction between stimulus type and test probe, *F*(1, 23) = .628, *MSE* = 0.017, *p* = .628, *η*^2^_*p*_ = .436.

A 2 (stimulus type: pictures vs sounds) × Probe (exemplar foil, novel foil) repeated measures ANOVA was conducted on proportion of G responses. There was a significant main effect of stimulus type, *F*(1, 23) = 6.61, *MSE* = 0.017, *p* = .017, *η*^2^_*p*_ = .223. There was a significantly lower proportion of guess responses for pictures (*M* = 0.017, *SE* = 0.021) compared with sounds (*M* = 0.143, *SE* = 0.018). There was no significant main effect of test probe, *F*(1, 23) = 2.19, *MSE* = 0.005, *p* = .152, *η*^2^_*p*_ = .087. There was no significant interaction between stimulus type and test probe, *F*(1, 23) = 3.56, *MSE* = 0.008, *p* = .072, *η*^2^_*p*_ = .134.

### Bayesian analysis

For Experiment 1B, in terms of the Bayesian analysis on proportion of remember responses, we found the evidence for the interaction was 3,228.23 (interaction)/1,342.93 (main effects) = 2.40. Thus, there was anecdotal evidence in support of the interaction. In terms of the Bayesian analysis on proportion of know responses, we found the evidence for the interaction was 0.430 (interaction)/1.01 (main effects) = 0.426. Thus, there was no evidence in support of the interaction. Finally, in terms of the Bayesian analysis on proportion of guess responses, we found the evidence for the interaction was 3.38 (interaction)/1.58 (main effects) = 2.14. Thus, there was anecdotal evidence in support of the interaction.

## Discussion

The accuracy results from Experiment 1B showed that the recognition advantage for pictures compared with sounds observed in the yes/no recognition test of Experiment 1A is also present in a 2AFC test. The fact that participants showed lower accuracy for sounds compared with pictures in both the exemplar and the novel test conditions suggests that the superior recognition advantage for pictures compared with sounds is due to the participant’s ability to encode and retrieve more verbatim and gist information for pictures compared with sounds. The results from the analysis of RKG judgements provides some support to the suggestion that the higher recognition memory for pictures compared with sounds was due to the participant relying more on recollection for remembering pictures compared with sounds, whereas participants relied more on familiarity for remembering sounds. The significantly higher proportion of remember responses for correctly recognised pictures follows with evidence provided by Dewhurst and Conway (1994) and Rajaram (1996) that the PSE is seen in higher proportions of remember compared with know responses in accurate recognition of pictures compared with words.

## Experiment 2A

Experiments 2A and 2B were designed to determine if providing written labels could promote better encoding of sounds such as the word “Windchimes” presented with the sound of chimes blowing in the wind. In the auditory domain, two classic studies showed labelling increased recognition memory for sounds (Bower & Holyoak, 1973; Clark et al., 1974). Bower and Holyoak (1973) showed recognition memory was higher for studied sounds given old labels during testing than those receiving new labels at test. Clark et al. (1974) found recognition memory was improved for labelable compared to non-labelable sounds and proposed that a dual-coding process occurred for labelable sounds. Thus, both Bower and Holyoak (1973) and Clark et al. (1974) showed the importance of labels for pictures and sounds in improving recognition accuracy during the test phase. However, Cohen et al. (2009) showed that presenting written labels for sounds during the encoding phase did not reduce the recognition advantage for pictures. We provided encoding and retrieval support for sounds by providing written labels during the study phase (Experiment 2A) or during both the study and test phases (Experiment 2B).

In Experiment 2A, we wanted to examine if providing verbal labels for sounds during the study phase would improve recognition memory in a yes–no test for sounds. However, the degree of specificity of the verbal label of the sound could also influence recognition memory. Previous research with pictures as stimuli, has shown that the type of label whether it be general or specific can influence the retrieval of the labelled studied item. In a study by Jörg and Hörmann (1978), participants were presented with pictures of objects to study for a later recognition test. Prior to presentations of the drawings, pictures were either presented with a general (e.g., flower) compared to a specific label (e.g., tulip). During the test phase, participants were presented very similar to dissimilar version of the studied drawings. The researchers found that with a general label, participants were able to distinguish versions that were dissimilar to the studied drawing, whereas participants were able to distinguish versions that were very similar to studied drawings if the label was specific. The researchers concluded that the generality or specificity of the verbal labels influenced which versions of the drawings were previously seen.

We predicted that written labels provided for sounds during the encoding phase would improve recognition memory for sounds. Participants viewing written labels with sounds would be able to appreciate the name of each sound. From a dual-coding perspective, this would allow participants to encode sounds in both perceptual and verbal codes. The written labels could also promote deeper semantic processing of the sounds and, from the perspective of fuzzy-trace theory, would increase the gist-based representation of the studied sounds. We based this prediction on the assumption that written labels could enable a dual coding of the sound. Participants viewing written labels with sounds would be able to associate the verbal label with the sound, which would promote deeper semantic processing and enable the storage of a more gist-based representation of the studied sound. To note, our written labels for sounds were in majority in a general form (e.g., verbal label of “Bell” for sound of church bell) rather than specific form (e.g., verbal label of “Basketball swish” for the sound of the swish of a basketball through a net). We predicted, therefore, that the recognition of sounds and pictures would be similar in the novel foil condition.

## Methods

### Participants

Twenty-two undergraduate (*Mean age* = 19.4, *SD* = 1.14; 12 females) students participated for course credit.

### Materials

The stimuli were the same as in Experiment 1A.

### Procedure

The method was the same as Experiment 1A, with the exception that written labels were presented for sounds during the study phase. For the 5 s duration that the sound was played, the label was shown in the centre of the screen in black Times New Roman size 40 font.

## Results

### Accuracy

The proportion of correct responses for each test probe are shown in Table 1. A 2 (stimulus type: picture vs sounds) × 3 (test probe: targets, exemplar foil, novel foil) repeated measures ANOVA conducted on these proportions revealed a significant main effect of stimulus type, *F*(1, 21) = 35.6, *MSE* = 0.012, *p* < .001, *η*^2^_*p*_ = .629. Higher accuracy was shown for pictures (*M* = 0.85, *SE* = 0.016) compared with sounds (*M* = 0.74, *SE* = 0.017). There was also a significant main effect of test probe, *F*(2, 42) = 65.8, *MSE* = 0.014, *p* < .001, *η*^2^_*p*_ = .758. Higher accuracy was shown for novel foils (*M* = 0.93, *SE* = 0.012) compared with targets (*M* = 0.81, *SE* = 0.019), with lowest accuracy for exemplar foils (*M* = 0.64, *SE* = 0.027). Pairwise comparisons showed all mean differences to be significant, *p* < .001. There was a strong trend for an interaction between stimulus type and test probe, *F*(2, 42) = 2.78, *MSE* = 0.014, *p* = .07, *η*^2^_*p*_ = .117. Paired sample *t*-tests showed that the mean difference in hit rates between target pictures and target sounds was significantly larger, *t*(21) = 4.39, *M* = 0.18, *p* < .001, than the mean difference of correct rejections for the exemplar foils, *t*(21) = 2.66, Mean difference = 0.10, *p* = .015, and novel foils, *t*(21) = 2.64, Mean difference = 0.06, *p* = .013.

#### Bayesian

We found the evidence for the interaction was 1.000 (interaction)/0.456 (main effects) = 2.19. Thus, there was anecdotal evidence in support of the interaction.

### Experiment 1A vs Experiment 2A comparison

To determine whether presenting written labels for sounds improved recognition performance for sounds, the results of Experiment 2A were compared to Experiment 1A. A (stimulus type: picture vs sounds) × 2 (test probe: old, exemplar foil, novel foil) × 2 (Experiment: 1A vs 1B) mixed factor ANOVA was conducted on proportion of correct responses with the between-subject factor being Experiment. There was no significant main effect of Experiment, *F*(1, 42) = 0.338, *MSE* = 0.031, *p* = .564, *η*^2^*p* = .008. There was a significant main effect of stimulus type, *F*(1, 42) = 86.3, *MSE* = 0.949, *p* < .001, *η*^2^_*p*_ = .673, with higher accuracy for pictures (*M* = 0.86, *SE* = 0.011) compared with sounds (*M* = 0.74, *SE* = 0.014). There was also a significant main effect of test probe, *F*(2, 84) = 114.89, *MSE* = 0.013, *p* < .001, *η*^2^_*p*_ = .732. Highest accuracy was shown for novel foils (*M* = 0.93, *SE* = 0.01) followed by targets (*M* = 0.83, *SE* = 0.01), and lastly exemplar foils (*M* = 0.64, *SE* = 0.02) as supported by Bonferroni pair-wise comparisons, *p* < .001. All interactions were not significant. In particular, the interaction between stimulus type and experiment was not significant, *F*(1, 42) = 0.204, *MSE* = 0.011, *p* = .654, *η*^2^_*p*_ = .005. The interaction between test probe and experiment was not significant, *F*(2, 84) = 0.808, *MSE* = 0.016, *p* = .449, *η*^2^_*p*_ = .019. The interaction between stimulus and test probe was not significant, *F*(2, 84) = 1.42, *MSE* = 0.017, *p* = .246, *η*^2^_*p*_ _=_ .033 and the interaction between stimulus type, test probe, and experiment was not significant, *F*(2, 84) = 1.09, *MSE* = 0.017, *p* = .343, *η*^2^_*p*_ _=_ .025.

### D prime

In Table 5, *d* prime (*d*′) estimates are shown for discrimination of target from foil and target from exemplar foil for pictures and sounds in Experiment 2A.

**Table 5.** Mean *d*′ and Criterion estimates are shown for discrimination of target from foil and target from exemplar foil for pictures and sounds in Experiments 2A and 2B.

Experiment	Stimulus type	*d*′ target–novel foil	*d*′ target–exemplar foil	C target–novel foil	C target–exemplar foil
Exp 2A	Picture	2.94 (0.53)	1.88 (0.69)	0.14 (0.23)	−0.38 (0.23)
	Sounds	2.00 (0.55)	0.93 (0.67)	0.33 (0.38)	−0.20 (0.41)
Exp 2B	Picture	2.69 (0.63)	1.60 (0.84)	0.26 (0.28)	−0.28 (0.31)
	Sounds	2.59 (0.79)	0.76 (0.74)	0.18 (0.26)	−0.73 (0.44)

Standard deviations of the means are given in parentheses.

A 2 (stimulus type: picture vs sounds) × 2 (test type: target–new foil vs target–exemplar foil) repeated measures ANOVA was conducted on *d*′ estimates. There was a significant main effect of stimulus type, *F*(1, 21) = 39.73, *MSE* = 0.49, *p* = .000, *η*^2^_*p*_ = .654. The mean *d*′ score was higher for pictures (*M* = 2.40, *SE* = 0.125) compared with sounds (*M* = 1.47, *SE* = 0.120). There was also a significant main effect of test type, *F*(1, 21) = 195.64, *MSE* = 0.128, *p* = .000, *η*^2^_*p*_ = .903 as *d*′ was greater for the target–novel foil condition (*M* = 2.47, *SE* = 0.091) than the target–exemplar foil condition (*M* = 1.40, *SE* = 0.117). The interaction between stimulus type and test type was not significant, *F*(1, 21) = 0.035, *MSE* = 0.068, *p* = .852, *η*^2^_*p*_ = .002.

#### Bayesian

We found the evidence for the interaction was 5.066 × 10^12^ (interaction)/1.498 × 10^13^ (main effects) = 0.338. Thus, there was no evidence in support of the interaction.

### Criterion

In Table 5, criterion estimates are shown for discrimination of target from novel foil and target from exemplar foil for pictures and sounds in Experiment 2A. A 2 (stimulus type: picture vs sounds) × 2 (*d*′ type: target–novel foil vs target–exemplar foil) repeated measures ANOVA was conducted on criterion correct responses. There was no significant main effect of stimulus type, *F*(1, 21) = 3.33, *p* = .08, *MSE* = 0.233, *η*^2^_*p*_ = .137. There was a significant main effect of *d*′ type, *F*(1, 21) = 195.64, *p* < .001, *MSE* = 0.032, *η*^2^_*p*_ = .903. Mean criterion estimates were more positive for target–novel foils (*M* = 0.238, *SE* = 0.042) than target–exemplar foils (*M* = –0.296, *SE* = 0.048). The interaction between stimulus type and *d*′ type did not approach significance, *F*(1, 21) = 0.035, *MSE* = 0.017, *p* = .852, *η*^2^_*p*_ = .002.

### Confidence

Refer to Table 3 and the online Supplementary Material containing analyses. There was higher confidence shown for pictures than sounds. Highest confidence was shown for targets, followed by novel foils. Lowest confidence in correct responses was shown for exemplar foils.

## Discussion

In Experiments 1A and 1B, one could argue that participants may have not been able to identify some sounds leading to higher recognition memory for pictures compared with sounds. In Experiment 2A, written labels were presented with sounds to address this issue. Interestingly, the presence of written labels with sounds during the study phase did not eliminate the recognition advantage for pictures. Written labels were provided for sounds during the study phase to promote deeper encoding, but a comparison of the proportion of correct responses for sounds between Experiments 1A and Experiment 2A indicates there was no benefit of the written labels at study. Our findings support Cohen et al. (2009), who found in their second experiment that written labels presented at encoding for sounds did not reduce or eliminate the higher accuracy for pictures compared with sounds.

## Experiment 2B

From the results of Experiment 2A, it was found that recognition memory for sounds was not improved with written labels being presented during encoding. We speculated that with written labels for sounds also provided at retrieval there would be a match between encoding and test, which would provide retrieval support for recognition memory for sounds. We predicted with written labels provided for sounds during the encoding and the test phases that recognition memory for sounds should be significantly improved and there would be an elimination of the recognition memory advantage for pictures in the novel test condition. This prediction was based on the finding by Bower and Holyoak (1973) who provided labelled sounds to participants and found higher recognition memory for old sounds, given their old labels at test compared to those receiving no or new labels at test.

## Methods

### Participants

Twenty-three undergraduate students (*Mean age* = 19.13, *SD* = 0.91; 13 females) participated for course credit.

### Materials

The stimuli were the same as in Experiment 1A.

### Procedure

The method was the same as Experiment 2A, with the exception that written labels were also presented for sounds during the test phase.

## Results

### Accuracy

The proportions of correct responses are shown in Table 1. A 2 (stimulus type: picture vs sounds) × 2 (test probe: target, exemplar foil, novel foil) repeated measures ANOVA revealed a significant main effect of stimulus type, *F*(1, 22) = 23.8, *MSE* = 0.016, *p* < .001, *η*^2^_*p*_ = .520, as accuracy was higher for pictures (*M* = 0.82, *SE* = 0.019) compared with sounds (*M* = 0.72, *SE* = 0.020). There was also a significant main effect of test probe, *F*(2, 44) = 108.72, *MSE* = 0.020, *p* < .001, *η*^2^_*p*_ = .832. Higher accuracy was shown for novel foils (*M* = 0.94, *SE* = 0.012) compared with targets (*M* = 0.84, *SE* = 0.025) with mean difference = 0.105, *p* = .001. There was lower accuracy for exemplar foils (*M* = 0.53, *SE* = 0.029) compared to novel foils, mean difference = 0.84, *p* = .025. Interestingly, there was a significant interaction between stimulus type and test probe, *F*(2, 44) = 23.40, *MSE* = 0.014, *p* < .001, *η*^2^_*p*_ = .51. Bonferroni pair-wise comparisons showed there was no significant difference in proportion of hits to picture and sound targets (mean difference = 0.030, *p* = .160). Moreover, there was no significant difference in proportion of correct rejections to picture and sound novel foils (mean difference = 0.014, *p* = .597). The interaction was due to a significantly higher proportion of correct responses for picture exemplar foils compared with sound exemplar foils (mean difference = 0.296, *p* < .001). A paired sample *t*-test showed that there was significantly higher correct rejection rate for picture exemplar foils compared with sound exemplar foils, *t*(22) = 5.71, mean difference = 0.296, *p* < .001.

### Bayesian analysis

For Experiment 2B, we found that the evidence for the interaction was 8.541 × 10^22^ (interaction)/3.049 × 10^15^ (main effects) = 28,012,463. Thus, there was substantial evidence for the interaction.

### D prime

In Table 5, *d*′ estimates are shown for discrimination of target from foil and target from exemplar foil for pictures and sounds. A 2 (stimulus type: picture vs sounds) × 2 (test type: target–novel foil vs target–exemplar foil) repeated measures ANOVA showed a significant main effect of stimulus type, *F*(1, 22) = 11.34, *MSE* = 0.440, *p* = .003, *η*^2^_*p*_ = .340. Discrimination was greater for pictures (*M* = 2.14, *SE* = 0.148) compared with sounds (*M* = 1.68, *SE* = 0.142. There was also a significant main effect of *d*′ type, *F*(1, 22) = 307.17, *MSE* = 0.159, *p* < .001, *η*^2^_*p*_ = .933 as the discrimination of target–novel foils (*M* = 2.64, *SE* = 0.130) was greater than the target–exemplar foils (*M* = 1.18, *SE* = 0.138). There was also a significant interaction between stimulus type and test type, *F*(1, 22) = 18.05, *MSE* = 0.178, *p* < .001, *η*^2^_*p*_ = .451. Bonferroni pair-wise comparisons showed *d*′ was significantly lower for sounds in the target–exemplar foil condition (*M* = 0.76, *SE* = 0.16) compared with pictures in the target–exemplar foil condition (*M* = 1.60, *SE* = 0.18), the mean difference being 0.840, *p* < .001. Yet, there was no significant difference in discrimination between picture target–novel foil (*M* = 2.68, *SE* = 0.13) and sound target–novel foil conditions (*M* = 2.59, *SE* = 0.18), mean difference = 0.092, *p* = .527.

### Bayesian analysis

We found the evidence for the interaction was 7.866 × 10^13^ (interaction)/1.230 × 10^11^ (main effects) = 639.5. Thus, there was substantial evidence for the alternative hypothesis as one would expect, given the traditional repeated measures.

### Criterion

Criterion estimates are shown for discrimination of target from novel foil and target from exemplar foil for pictures and sounds in Table 5. A 2 (stimulus type: picture vs sounds) × 2 (test type: target–novel foil vs target–exemplar foil) repeated measures ANOVA based on criterion estimates revealed a significant main effect of stimulus type, *F*(1, 22) = 14.37, *MSE* = 0.117, *p* = .001, *η*^2^_*p*_ = .395. A less negative criterion was shown for pictures (*M* = –0.009, *SE* = 0.058) compared with sounds (*M* = –0.28, *SE* = 0.066). There was a significant main effect of test type, *F*(1, 22) = 307.17, *MSE* = 0.040, *p* < .001, *η*^2^_*p*_ = .933. Mean criterion was more positive for target–novel foils (*M* = 0.219, *SE* = 0.046) than target–exemplar foils (*M* = –0.509, *SE* = 0.063). There was also a significant interaction of stimulus type and test type, *F*(1, 22) = 18.05, *MSE* = 0.044, *p* = .000, *η*^2^_*p*_ = .451. Paired sample *t*-test showed that there was a significantly more positive criterion shown for pictures in the target–exemplar foil condition compared with sounds, *t*(22) = 4.60, *p* < .001. In contrast, there was similar criterion shown for pictures and sounds in the target–novel foil condition, *t*(22) = 0.643, *p* = .527.

### Confidence

Mean confidence judgements for correct responses are shown in Table 3. Analysis of confidence is shown in the online Supplementary Material. Although there was no difference in mean confidence in correct responses to pictures and sounds, there was higher confidence shown for targets compared with novel foils. In contrast, similar confidence in correct responses was shown for exemplar and novel foils.

## Discussion

Presenting written labels at both study and test increased the hit rate for sounds to the level of the hit rate for pictures. There was still, however, a much higher false alarm rate to exemplar foils for sounds compared with pictures. The presentation of written labels for sounds, both during encoding and test eliminated the recognition advantage for pictures when considering hit rate for targets and correct rejection rate for novel foils. However, with exemplar foils, there was still a higher correct rejection rate for pictures compared with sounds. This pattern of results indicates that the presentation of labels for sounds during encoding and test increased the encoding and retrieval of gist information to a similar level as pictures. However, the presence of labels during study and test phase did not benefit the encoding and retrieval of relevant verbatim details for correct rejection of exemplar foils. We suggest that the written labels at study and test may have promoted a more gist-based and less verbatim-based representation, which benefitted the discrimination of targets and novel foils but decreased the discrimination of targets and exemplar foils.

## Experiments 3A and 3B

The previous experiments showed recognition memory for pictures to be higher than sounds and this recognition advantage occurred because of participants encoding and retrieving more perceptually detailed and gist-based representations of the pictures. In contrast, the encoding and retrieval of sounds was less perceptually detailed and less gist-based unless written labels were presented with sounds at study and test. One explanation for these findings could be that pictures are perceived by participants as less similar to each other than sounds, which would account for the higher recognition of pictures compared with sounds. The reduced similarity of the pictures would occur if pictures were distinctively different from each other based on perceptual details, which are characteristic of verbatim information. Gloede and Gregg (2019) using a perceptual discrimination task, found that participants showed equal perceptual discrimination for pictures and sounds, suggesting that pictures and sounds have a comparable degree of perceptual similarity. In their perceptual discrimination task, pairs of stimuli were sequentially presented. The pairs were either different as in an exemplar pair (e.g., dog A and dog B) or similar (e.g., dog A and dog A) as in paired with itself. Participants were to press a key to indicate “Same” or “Different.”

However, the degree of subjective similarity of pictures and sounds can also be measured with the use of similarity judgements on a rating scale. Gygi et al. (2007) investigated the acoustical correlates of similarity and categorisation judgements of environmental sounds. In Experiment 1, participants rated the similarity of sounds on a scale of 1 to 7 (not similar to as similar as possible) to compare a large list of environmental sounds. A three-dimensional scaling (MDS) solution was implemented and showed clustering of sounds by type of source, such as vocalisations, impacts, and water sounds. Melara et al. (1992) investigated the nature of similarity relations among three pairs of interacting dimensions: (1) the integral dimensions of auditory pitch and loudness, (2) the configural dimensions as in each member of pair facing left or right, and (3) the cross-modally corresponding dimensions of visual position and auditory pitch (e.g., high pitch and high position). Participants rated similarity of sounds, but also provided a similarity rating on auditory pitch and loudness. They found that instructions to rate overall similarity encouraged participants to attend to stimuli as a whole and led to Euclidian rule in similarity scaling. In contrast, instructions to focus on dimensions led subjects to consider each stimulus dimension separately.

Thus, judging similarity for pictures and sounds requires basing similarity on different features related to stimulus dimensions. We felt an overall rating of similarity of pictures and sounds would not provide a detailed understanding of the qualitative difference in similarity. An individual can base similarity on two dimensions, perceptual and semantic features that represent perceptual and gist information, respectively. For pictures, we determined that semantic features in the form of category and function and perceptual features in the form of colour and shape would be essential for discriminating targets from lures. For sounds, we determined that semantic features in the form of category and function and perceptual features in the form of pitch, pattern, and loudness would be essential for discriminating targets from lures. We integrated these assumptions into the development of a procedure where participants would indicate which semantic feature (i.e., category, function) and perceptual feature (i.e., colour and shape for pictures; loudness, pitch, and pattern for sounds) they used to base their similarity judgements. Specifically, after providing a similarity rating, participants would provide two judgements to indicate what perceptual and semantic feature they based their similarity rating. To our knowledge, no researcher has collected qualitative information in such a way for a similarity rating judgement task.

One experiment was conducted with the above design. In Experiment 3A, participants provided their similarity judgements but also indicated which semantic and perceptual features they used to base each of their similarity judgements. Moreover, written labels were provided for sounds and pictures. We thought that providing labels would enable participants to make more informative decisions on rating of similarity of pictures and sounds. In Experiment 3B, no instructions on how to base similarity judgements and no labels were provided. We predicted that pictures would be rated less similar than sounds and this would account for the higher recognition memory for pictures compared with sounds. We made this assumption since several studies have shown that distinctiveness or reduced similarity within a stimulus group leads to higher recognition memory for the individual stimuli (Brady et al., 2008; Konkle et al., 2010a; Stark et al., 2019). Konkle et al. (2010b) showed that with an increase in the number of exemplars within a category, there was a decline in scene recognition in the exemplar test condition. Stark et al. (2019), using a mnemonic similarity task (MST), showed that as the similarity of lures to targets increased, there was a noticeable decline in participant’s ability to distinguish between targets and lures.

## Methods

### Participants

Twenty undergraduate students (*Mean age* = 19, *SD* = 1.08; 12 females) participated for course credit in Experiment 3A and 20 participants (*Mean age* = 19.1, *SD* = 1.02; 11 females) in Experiment 3B.

### Materials and procedure

**Figure 2.**
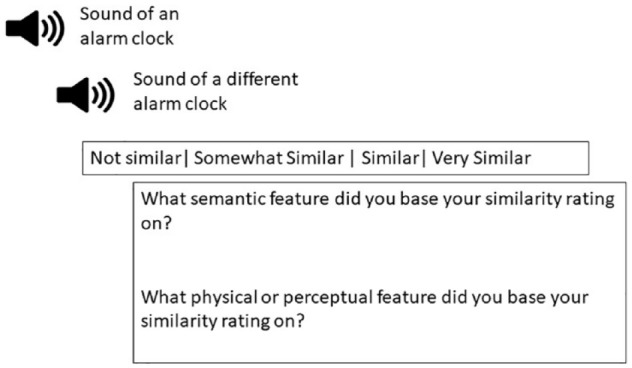
Displayed is the setup of similarity ratings for Experiment 3A and 3B. Note, not shown in figure are the labels and the drop-down box. Refer to the online Supplementary Material 2 for the list of items with labels presented during the test phase. Note for Experiment 3B, labels were not provided, and participants did not indicate what perceptual and semantic feature they based their similarity rating. Figures of fixation boxes with interstimulus interval (ISI) of 250 ms are not included.

The exact set of picture and sound pairs (i.e., novel and exemplar pairs) presented in Experiment 1B were presented in Experiment 3A. Figure 2 displays the general paradigm for Experiment 3A. The procedure where the participant met with the experimenter was similar to previous experiments with the exception that participants underwent a practice session to understand how to judge the similarity of pictures and sounds.

After meeting with the experimenter, the participant then started the actual online experiment hosted on the Gorilla.sc platform. On the computer screen, participants were shown instructions on how to base their rating of similarity between the pictures or sounds. The following is a summary of the instructions. The full set of instructions is provided in supplemental information. Participants were told to base their rating of similarity between the pictures or sounds on the number of features that were shared between the pictures or sounds (i.e., category, function, or physical properties such as colour and shape or loudness, pitch and pattern). Moreover, they were told that the degree of similarity between pictures or sounds could be determined by the number of features shared between the pictures or shared between the sounds. That is, no common features (i.e., *not similar*), one common feature (i.e., *somewhat similar*), two common features (i.e., *similar*), and three or more common features (i.e., *very similar*). They were also told that the choice of “none” was to be selected when their similarity rating was not based on any of the choice of features.

During the trial, a fixation cross was presented for 125 ms, after which a picture or sound would be presented for 5 s followed by fixation for 125 ms and then the second picture or sound would be displayed for 5 s. A screen then appeared for which the participant provided their similarity judgement of not similar, somewhat similar, similar, or very similar. If they had made a similarity judgement on two pictures, two questions appeared on the next screen. The questions were, “What semantic feature did you base your similarity rating on?” and “What physical or perceptual feature did you base your similarity rating on?.” Participants indicated the feature they based their similarity ratings on by selecting from the choices presented in two drop-down boxes. One drop-down box was for the choice of semantic feature, the choices being category, function and none. The other drop-down box was for the choice of perceptual feature, the choices being colour, shape, and none. In contrast, if they had made a similarity judgement on two sounds, the choices provided in the drop-down box for perceptual features was different with the choices being pitch, pattern, loudness, and none.

In total, there were 26 picture trials and 26 sound trials. Of the 26 trials, 13 were trials where exemplars were presented and 13 were trials where novel items were presented. As a result, participants were to provide 26 similarity ratings for picture trials and 26 similarity ratings for sound trials. Moreover, for picture trials, participants provided 26 answers for semantic features and 26 answers for perceptual features. Similarly, for sound trials, they provided 26 answers for semantic features and 26 answers for perceptual features.

Materials and Procedure in Experiment 3B were the exact same as Experiment 3A with the exception that there were no labels for pictures and sounds and no instructions on how to base similarity judgements was provided.

## Results

### Experiment 3A

#### Overall mean similarity across conditions

Table 6 presents the relevant overall mean similarity ratings for the exemplar and novel foil conditions in Experiments 3A and 3B. For reference, the mean proportions of responses for each similarity rating (i.e., *very similar*, *similar*, *somewhat similar*, *not similar*) in each condition (i.e., exemplar foil, novel foil) for each experiment are presented in Table 7 and separate analyses with Tables 8–11 containing ratings for semantic and perceptual details, are provided in the online Supplementary Material.

**Table 6.** Overall mean similarity ratings for the exemplar and novel foil conditions in Experiments 3A and 3B.

Experiment	Stimulus type	Exemplar condition	Novel condition
Exp 3A	Picture	3.60 (0.36)	1.10 (0.13)
	Sound	2.50 (0.70)	1.36 (0.34)
Exp 3B	Picture	3.38 (0.44)	1.18 (0.26)
	Sound	2.93 (0.54)	1.39 (0.30)

Standard deviations of the means are given in parentheses.

A 2 (stimulus type: picture vs sounds) × 2 (condition: exemplar vs novel) repeated measures ANOVA was conducted on the proportions of mean similarity ratings. There was a significant main effect of stimulus type, *F*(1, 19) = 22.8, *MSE* = 0.150, *p* = .000, *η*^2^_*p*_ = .546. Similarity was rated as higher for pictures (*M* = 2.34, *SE* = 0.038) compared with sounds (*M* = 1.93, *SE* = 0.094). There was a significant main effect of condition, *F*(1, 19) = 243.6, *MSE* = 0.282, *p* = .000, *η*^2^_*p*_ = .925; similarity was rated higher in the exemplar (*M* = 3.05, *SE* = 0.107) compared to the novel (*M* = 1.22, *SE* = 0.045) condition. There was also an interaction between stimulus type and condition, *F*(1, 19) = 138, *MSE* = 0.067, *p* < .001, *η*^2^_*p*_ = .880. Paired sample *t*-tests showed that there was a higher mean similarity rating for pictures (*M* = 3.60, *SE* = 0.08) compared with sounds (*M* = 2.50, *SE* = 0.16) presented in the exemplar condition, *t*(19) = 8.49, *p* < .001. In contrast, there was a higher similarity rating for sounds (*M* = 1.36, *SE* = 0.08) compared with pictures (*M* = 1.10, *SE* = 0.03) in the novel condition, *t*(19) = 3.57, *p* = .002.

#### Bayesian analyses

We found the evidence for the interaction was 6.84 × 10^21^ (interaction)/1.40 × 10^10^ (main effects) = 4.89 × 10^11^. Thus, there was substantial evidence for the alternative hypothesis, as one would expect given the traditional repeated measures.

### Discussion

The results of Experiment 3A showed that mean similarity was higher for pictures than sounds depending on the condition. In the exemplar foil condition, pictures were rated more similar than sounds, whereas in the novel condition, sounds were rated more similar than pictures. In terms of the analysis of selection of features to base their similarity ratings presented in Supplementary Material, it was apparent that participants were biased to certain semantic and perceptual features to base their similarity rating. There was higher mean similarity based on category than function for both pictures and sounds. In terms of perceptual features, there was a higher mean similarity rating based on colour than shape for pictures, whereas for sounds there was higher mean similarity ratings based on pattern than loudness.

Based on the findings, it seems that participants were able to base similarity of pictures and sounds on semantic and perceptual features, which indicates that there were similar number of features for pictures and sounds. The fact that participants indicated higher similarity rating for pictures than sounds in exemplar condition, suggests that in the exemplar foil condition, there were more matching perceptual features for pictures compared with sounds, whereas in the novel foil condition, pictures had more distinguishing perceptual features compared with sounds.

## Experiment 3B

### Mean similarity across conditions

Relevant means are in Table 6. A 2 (stimulus type: picture vs sounds) × 2 (condition: exemplar, novel) repeated measures ANOVA was conducted on mean similarity ratings. There was no significant main effect of stimulus type, *F*(1, 19) = 8.45, *MSE* = 0.033, *p* = .009, *η*^2^_*p*_ = .308. As expected, there was a significant main effect of condition, *F*(1, 19) = 246.7, *MSE* = 0.284, *p* < .001, *η*^2^_*p*_ = .928. Similarity was rated higher in the exemplar (*M* = 3.15, *SE* = 0.10) compared to the novel (*M* = 1.29, *SE* = 0.05) condition. There was also an interaction between stimulus type and condition, *F*(1, 19) = 35.8, *MSE* = 0.060, *p* < .001, *η*^2^_*p*_ = .653. Paired sample *t*-tests showed that the mean similarity rating for pictures (*M* = 3.38, *SE* = 0.10) was greater than sounds (*M* = 2.93, *SE* = 0.12) in the exemplar condition, *t*(19) = 6.17, *p* = .002. In contrast, there was higher similarity rating for sounds (*M* = 1.39, *SE* = 0.07) compared with pictures (*M* = 1.18, *SE* = 0.06) in the novel condition, *t*(19) = 3.29, *p* = .004. Despite the absence of written labels and specific rating instructions, the results of Experiment 3B replicated the pattern of similarity ratings across conditions seen in Experiment 3A.

#### Bayesian analyses

We found the evidence for the interaction was 1.66 × 10^15^ (interaction)/3.96 × 10^9^ (main effects) = 419,191. Thus, there was substantial evidence for the alternative hypothesis, as one would expect given the traditional repeated measures.

## Discussion

Over two experiments, we found a consistent pattern of higher similarity ratings provided for pictures compared with sounds in the exemplar foil condition, but higher similarity ratings provided for sounds in the novel foil condition. In both Experiments 3A and 3B, participants were to provide a similarity rating for pairs in an exemplar and novel foil condition. However, in Experiment 3B, there were no labels and no instructions for providing similarity rating based on semantic and perceptual features. Our main finding was that in two experiments that differed on labels being presented for sounds and the presence of instructions to base similarity on perceptual and semantic features, mean similarity was shown to be higher for pictures than sounds depending on the condition. That is, in the exemplar foil condition, pictures were rated more similar than sounds, whereas in the novel condition, sounds were rated more similar than pictures. This finding was unexpected as we predicted that since there was higher recognition memory for pictures compared with sounds as found in earlier experiments, there would be, in general, less similarity within pictures compared with sounds. In the general discussion, we propose that the above finding shows that categorisation of pictures is more precise than that of sounds due to there being more features for pictures than for sounds.

## General discussion

The purpose of this study was to examine the mechanism for the recognition memory advantage for pictures compared with sounds as shown by several researchers (Bigelow & Poremba, 2014; Cohen et al., 2009, 2011; Gloede et al., 2017; Gloede & Gregg, 2019; Kassim et al., 2018). Several explanations for the recognition memory advantage for pictures compared with sounds have been suggested. Gloede and Gregg (2019) have provided evidence that auditory memory representations are coarser and gist-based, whereas visual memory representations are more fine-tuned, specific, and detailed. Other explanations point to the nature of encoding of pictures and sounds leading to this difference in the retrieved memory representation. The distinctiveness of pictures could have led to the formation of more precise memory representation retrieved during the test phase. Distinctiveness can be due to perceptual or conceptual distinctiveness. Pictures may be more perceptually distinctive than sounds due to the nature of presentation of pictures compared with sounds. As noted by Gloede and Gregg (2019), visual features of objects such as colour and shape are simultaneously available, whereas auditory features such as the loudness and the pitch of objects unfold over time. Thus, visual information can convey a large amount of perceptual information in a single glance.

However, pictures may be more conceptually distinctive than sounds for a variety of reasons. Pictures are more likely than sounds to be represented in both a verbal and an imaginal form following Paivio’s dual-coding theory. Pictures could also be more conceptually distinctive than sounds due to meaning. Pictures may receive more semantic processing than sounds; deeper or more conceptual processing has been identified as a mechanism to account for higher associative recognition of pictures than words pairs (Hockley, 2008).

Alternatively, it could be due to the physical nature of the presentation of the pictures and sounds that pictures are more conceptually and perceptually distinctive than sounds. As discussed by Ensor, Bancroft, and Hockley (2019) and Gloede and Gregg (2019), pictures are processed spatially with participants able to move visually from one feature to another, whereas sounds must be processed temporally. Ensor, Bancroft, and Hockley (2019) suggest that due to the temporal distribution of features present in sounds, participants cannot spend more time on one sound feature than another^6^. In the case of the sound of the bell, the participant is not exposed to all features of the sound for the entire duration of 5 s. For a picture of an object such as a bell, when viewing the picture, a person can move their eyes from one feature to another. Due to the difference in processing of sounds and pictures, more information can be processed for pictures in a shorter period.

Finally, conceptual distinctiveness for pictures could be greater than sounds, due to the nature of mapping of the physical presentation onto an exemplar of the category. For example, presenting a picture of a clock or bird would map easily onto a specific or subordinate exemplar of the category, but because sounds do not have the same perceptual specificity, they are likely mapped onto a basic level or superordinate category level representation. Hearing a bird chirping tells you it is a bird, but does not tell you which type of bird, unless you are an expert on bird calls. Similarly, hearing an engine running may indicate that it is a vehicle, but not the type of vehicle.

Six experiments were conducted to determine the mechanism for the recognition memory advantage for pictures compared with sounds. First, yes/no and 2AFC recognition were compared to evaluate the verbatim and gist representations of pictures and sounds. Second, the effect of providing written labels on the recognition of sounds was examined. Finally, differences in the conceptual and perceptual similarity between pictures and sounds were evaluated.

Yes/no and 2AFC recognition for verbatim and gist representations were compared in Experiments 1A and 1B. Gloede and Gregg (2019) used a yes/no recognition test to show verbatim information is greater for retrieved pictures than sounds. However, the 2AFC test as shown by Ahmad et al. (2017), provides a more direct measure of both the retrieval of gist and verbatim information. Accuracy in the exemplar test condition reflects retrieval of verbatim information, whereas accuracy in the novel test condition reflects the availability of gist information. In Experiment 1A, both pictures and sounds were presented during the study phase and yes/no recognition memory was tested. Participants showed higher recognition memory for pictures compared with sounds. Interestingly, this recognition advantage was shown for three types of test probes: targets, related exemplar foils, and unrelated novel foils demonstrating a mirror effect. Gloede and Gregg (2019) also showed lower recognition memory for sounds compared with pictures for exemplar foils. Our interpretation of results was similar to that of Gloede and Gregg (2019) with the exception that we found that there was reduced retrieval of both gist and verbatim information relevant to studied sounds as shown statistically in the nonsignificant interaction between stimulus type and test probe, which was confirmed by our Bayesian analysis.

This contrast in findings may have been present due to difference in study presentation format. Whereas Gloede and Gregg (2019) presented equivalent pictures (e.g., picture of horse) and sounds (e.g., sound of horse) in two separate study blocks that was followed by the test phase, we presented different counterbalanced pictures and sounds during the study and test phases. Thus, it would have been easier for participants to retrieve studied and correctly reject exemplar foil sounds in the study by Gloede and Gregg (2019) compared with our Experiment 1A. However, it should be noted that there is varied opinion on the significance of a *p*-value of .049 supporting a significant interaction, as it is close to *p* = .05 which is not significant. If one were to have the opinion that the interaction is not significant, we suggest that Experiment 1A findings by Gloede and Gregg (2019) are largely consistent with the view that more verbatim and gist information were retrieved for pictures than sounds, rather than only verbatim information.

Supporting this interpretation of the results from Experiment 1A, participants showed higher recognition for pictures compared with sounds in forced-choice comparisons of the exemplar and novel test conditions in Experiment 1B. The results provide support for fuzzy-trace theory in that the representation retrieved for pictures is both more perceptually detailed and includes more gist information compared with that of sounds. The fact that accuracy was higher in the novel condition for pictures compared with sounds suggests that both a more verbatim and a more gist-based representation is retrieved for pictures than for sounds when accurate recognition takes place. As Ensor, Bancroft, and Hockley (2019) noted, participants cannot devote more time to some features of sounds over other features because sounds are distributed temporally rather than spatially. Due to sounds being encoded temporally, there is less opportunity to encode gist and perceptual details. It should be noted that one would predict according to the fuzzy-trace theory that to discriminate a target from an exemplar foil in a 2AFC recognition test, a stored verbatim representation of the target in LTM that supports recollection would be required, whereas one would predict according to the Complementary Learning Systems model (CLS; Migo et al., 2009; Norman & O’Reilly, 2003) that familiarity alone can support performance when the items are very similar.

The results of the remember and know procedure, indicated that participants showed more remember responses for pictures than sounds suggesting recollection was relied on more than familiarity for remembering pictures than sounds. This difference in reliance on recollection for retrieval from memory could account for the superior recognition memory of pictures. The relation between recollection and familiarity to the retrieval of verbatim and gist traces was suggested by Brainerd and Reyna (2002) in a section in their review article, under the heading titled Principle 5: Verbatim and Gist Processing Both Cause Vivid Remembering. According to Brainerd and Reyna (2002), recollection is a more vivid form of remembering, which is supported by the retrieval of verbatim traces, whereas the retrieval of gist traces supports a generic form of remembering, sometimes called familiarity. Thus, the higher proportion of remember responses in correct recognition of pictures compared to sounds may support the view that more verbatim information is retrieved in correct recognition of pictures compared with sounds. Recollected pictures may also contain sensory perceptual attributes, which is a form of verbatim information that is not present in memory traces of sounds correctly retrieved from LTM. In fact, Dewhurst and Conway (1994) suggested that the greater proportion of recollective experiences associated with pictures compared with words could be due to sensory-perceptual attributes being present in retrieved pictures.

To confirm that the picture recognition advantage over sounds is due to the retrieval of both more gist and verbatim information, we examined the effects of written labels on recognition memory for sounds compared with pictures on the assumption that written labels would encourage encoding of a more gist-based representation for sounds and result in similar discrimination for pictures and sounds compared with novel foils. In Experiment 2A, written labels were presented at encoding for sounds. Written labels would enable a dual coding of the sound. Participants viewing written labels with sounds would be able to associate the verbal label with the sound, which would promote deeper semantic processing and enable the storage of a more gist information of the studied sound. We found, however, that there was still higher recognition memory for pictures compared with sounds. The presence of labels during study phase did not improve recognition memory for sounds during the test phase. A future direction could be to examine whether instructing participants to generate their own written labels would promote a deeper level of encoding that would lead to reduction in the PSE. Rabinowitz et al. (1982) found specific retrieval cues aided retrieval of stored information better than general cues. Perhaps generating ones’ own written labels would enable storage of a specific verbal retrieval cue.

In Experiment 2B, labels for sounds were provided both during study and test based on the assumption that if written labels were also provided at retrieval there would be a match between encoding and test, which would provide retrieval support for recognition memory for sounds. Interestingly, hit rates for target pictures and target sounds were similar. However, there was lower discrimination of sound exemplar foils compared with picture exemplar foils. In fact, we found a significant interaction between stimulus type and test probe. The written labels presented during study phase and test phase may have promoted a less verbatim and more-gist based representation of sounds, which benefitted the discrimination of sound target and novel foils but decreased discrimination of sound exemplar foils from studied sounds.

Lupyan (2008) proposed that labels promote categorical representations through top-down processing. In Lupyan’s study, pictures of chairs and lamps were presented. For half of the list, participants were to label the items as a chair or lamp and in the other half of the list participants were to provide a preference rating of each item. During the test phase, participants were presented with studied pictures of chairs and lamps and new pictures of chairs and lamps. When participants classified using a verbal label compared to providing a preference rating, participants showed lower recognition memory performance for both targets and related exemplars of familiar items. Lupyan suggested that lower hit rates for items classified with category labels arise from a representational mismatch caused by top-down effects of the label. That is, when category labels are activated, they produce top-down feedback that activates features stored with the category on previous occasions, which would cause difficulty in discriminating studied and non-studied items of the same category.

As expected, we found participants showed higher confidence in correct responses in their recognition of pictures compared with sounds in both Experiment 1A and Experiment 2. Due to the exclusion of participants who provided inconsistent ratings of confidence, we cannot attribute this higher confidence due to retrieval of more verbatim and gist information for pictures compared with sounds. We believe the inconsistent results could be attributed to certain participants not following instructions, because of the complexity of the choices for confidence ratings may have led to confusion or misinterpretation. A future direction would be to use a confidence rating scale as Ahmad et al. (2017) implemented in their study, where participants provided confidence rating on a scale of 1 to 3, 1—“*not sure*,” 2—“*sure*,” 3—“*very sure*” for old and new responses. Researchers should also examine if greater retrieval of gist information could also lead to higher confidence in accurate responses for pictures compared with sounds. Reliance on retrieval of gist representations can result in high confidence responses attributed to phantom recollection as shown with older adults who may fail to remember specific but not gist representations (Greene et al., 2022).

In Experiment 2B, we found an increase in hit rate for sounds that were provided with written labels rather than a decrease in hit rate to labelled pictures as Lupyan (2008) had found, as we also provided written labels during the test phase. In Experiment 2B, when the participant heard windchimes, the label of windchimes overrode the specific perceptual features of sound of windchimes that would be stored in LTM. Thus, when the label for the exemplar of windchimes was presented with the new sound during test phase, participants had difficulty discriminating the studied and new representation of the studied sound of windchimes (i.e., exemplar) based on memory of the perceptual details. In contrast, participants could distinguish between novel and target sounds based on memory for the labels or gist of the sounds. However, it is important to note that recognition memory for both novel and exemplar pictures was higher than that of sounds in Experiment 2A, even with labels presented for sounds, which supports the conclusion that pictures had both greater gist-based and verbatim-based representations than sounds.

It follows that pictures were processed using both top-down (conceptual) and bottom-up (perceptual) processing to a greater extent than sounds even when labels were presented for sounds only during the encoding phase, which was the case for Experiment 2A. Moreover, one could argue that participants in both Experiments 1A and 1B showed higher recognition memory for pictures compared with sounds because participants may not have been able to identify some of the sounds; yet even with written labels presented for sounds during encoding (Experiment 2A) and encoding and retrieval (Experiment 2B), there was higher recognition memory shown for pictures compared with sounds.

Experiments 3A and 3B were designed to test the view that the greater difference in similarity between pictures and sounds contributed to higher distinctiveness leading to higher recognition for pictures compared with sounds. Participants rated the similarity of pairs of pictures and pairs of sounds. These pairs were taken from the test pairs that were presented in the novel and exemplar test conditions of Experiment 1B. In Experiment 3A, participants were given instructions to base judgements of similarity on perceptual and semantic features; and in Experiment 3B, participants were not given specific instructions. In both experiments, pictures were rated more similar in the exemplar condition, whereas sounds were rated more similar in the novel test condition.

We suggest that there are more distinctive perceptual and conceptual features in pictures than sounds. In the exemplar condition, pictures have more matching or related features and are rated more similar and pictures have more unrelated features in the novel test condition and are rated less similar. As a result, sounds would be rated less similar in the exemplar condition and more similar in the novel condition. Consider two pictures of an airplane. As they share many similar conceptual and perceptual features, they would be rated as being very similar. However, if they have even one different feature (e.g., colour), then they would be easy to distinguish. That is, the features that are used to make similarity ratings are not the same features that are used to distinguish exemplar targets. Moreover, there could be many more similar features than distinguishing features if the distinguishing features are salient and encoded. In contrast, if sounds such as of two similar airplanes have fewer and more impoverished features, then there would be fewer shared features to support high similarity ratings, in addition to fewer encoded and retrieved distinguishing features to support recognition.

Interestingly, the results from Experiments 3A and 3B support the suggestion that categorisation of pictures is more precise than that of sounds due to there being more features in pictures than sounds, which led to more similar ratings for pictures than sounds. We would expect more similar ratings in the exemplar compared to the novel condition, because more features are similar in an exemplar condition and this result was shown to a greater extent for pictures compared with sounds. The lack of precision of categorisation of sounds was further supported by the finding of higher similarity for sounds than pictures in the novel condition, in both Experiments 3A and 3B.

Ensor, Bancroft, and Hockley (2019) argued that dual-coding theory provides the best account of the sound superiority effect that is observed in tests of free recall (Crutcher & Beer, 2011). In contrast, the finding of a picture superiority but not a sound superiority effect in tests of recognition can best be explained by considerations of distinctiveness. According to the physical distinctiveness account (Ensor, Surprenant, & Neath, 2019; Mintzer & Snodgrass, 1999; Weldon & Coyote, 1996), pictures vary more in their physical features than words. It follows that, in contrast to pictures, the distinctiveness of the perceptual features of sounds is not sufficient to confer a memory advantage for sounds over words. According to the conceptual distinctiveness account, picture identification involves identifying which perceptual features are diagnostic of its identity. This identification process induces a deeper level of processing and produces a more distinctive memorial representation that benefits LTM for pictures (Hamilton & Geraci, 2006). In contrast, sounds heard on their own (i.e., without other extraneous noises) consist only of diagnostic perceptual features and therefore the identification of sounds do not benefit from the deeper level of processing that pictures undergo (Ensor, Bancroft, & Hockley, 2019). In addition, observers can process the features of pictures strategically by focusing on some features more than others, whereas listeners must process auditory features serially as they are presented temporally rather than spatially.

This study addressed the question of distinctiveness for pictures and sounds in three ways. First, Experiments 1A and 1B showed that the memorial advantage for pictures over sounds is seen both when recognition can be based on conceptual information (or gist) in the novel test condition and when memory for perceptual details (or verbatim information) is required in the exemplar test condition that equates conceptual information. This supports the view that both perceptual and conceptual information are richer or more distinctive for pictures than sounds. Second, Experiments 2A and 2B show that providing conceptual information in the form of a verbal label that accompanies the sounds at study, or at both study and test, does not eliminate the recognition advantage of pictures over sounds. We suggest that gist and verbatim information jointly determine the PSE, meaning that only enhancing one of them is not sufficient to eliminate the PSE. The finding that providing written labels enhance hits for targets but not correct rejections of exemplar foils, is consistent with fuzzy-trace theory’s assumption that gist traces support both true and false memory, whereas verbatim traces support true memory and suppress false memory.

Finally, Experiments 3A and 3B showed that the target and lures were rated more similar in the exemplar condition compared to the novel test condition. This is not surprising as exemplars from the same category would share more similar features than exemplars from different categories. More surprising was that pictures were rated more similar in the exemplar condition than sounds even though pictures were recognised more accurately than sounds. This suggests that while overall the features of pictures were more similar, there were still more perceptual features that distinguished old and new exemplars for pictures than for sounds. In addition, sounds were rated more similar than pictures in the novel condition indicating that the perceptual features of sounds are not as differentiating as the features of pictures for exemplars from different conceptual categories.

However, our interpretation of findings from experiments conducted on similarity ratings for pictures and sounds should be taken as suggestive but not strong and direct evidence for the distinctiveness account. Methods that have been proven to measure distinctiveness to support the PSE or directly affect types of distinctiveness should be implemented to examine differences in recognition memory for pictures compared with sounds. For example, one could reduce conceptual distinctiveness of pictures by increasing the conceptual similarity between pictures within the study list and examine if there is a decrease in PSE. A similar manipulation was implemented by Konkle et al. (2020b), where the number of similar scene exemplars in the study list was increased. Alternatively, one could make sounds more perceptually distinctive by manipulating properties of the sound such as making it louder or increasing pitch and examine whether it influences the relationship between distinctiveness and recognition memory for sounds.

Moreover, a future direction would be to examine the differences in the way participants encode and retrieve pictures compared with sounds. It may be because the visual features of pictures are presented spatially and simultaneously, whereas sounds are presented in a temporal manner as in unfolding over time, which enables participant to pay more attention to different aspects of pictures than sounds. Consequently, participants may be better able to encode unique features of pictures compared with sounds, which aids in discriminating similar pictures or similar sounds to stored representations of target pictures or sounds.

Finally, we note another limitation in our research study. The degree of similarity shown for pictures or sounds of our studied items to some of our novel items raises the question that we may not have controlled for the level of specificity of memory representations being encoded in memory. Perhaps some of our stimuli may not have been categorically distinct or may have been perceived by participants as not categorically distinct from each other. For example, if the participants saw a picture or heard a sound of a soccer ball during the study list and were given a yes–no recognition test that included a picture of a studied soccer ball and a new picture of ping-pong ball, some degree of verbatim representation would be needed to reject the similar ping-pong ball. Indeed, Greene and Naveh-Benjamin (2020, 2022) have provided strong evidence for the “specificity principle” suggested by Surprenant and Neath (2009) and alluded to by suggestion of hierarchies of gist in semantic representations (Brainerd & Reyna, 2015) and in the existence of a continuum with no categorical break between semantic and episodic representations (Craik, 2002).

In their first experiment, Greene and Naveh-Benjamin (2020) showed that when tested on recognition of face–scene pairs by use of associative-recognition tests following variable delays, older adults were unable to retrieve more specific representations. Greene and Naveh-Benjamin (2020) suggested that retrieval of associations exists on multiple levels of specificity. They supported their findings with a later study manipulating degree of attention during encoding (Greene et al., 2022). Thus, future research should examine how level of specificity in terms of the verbatim representation could affect recognition memory for pictures and sounds. Interestingly, testing retrieval of source information at levels of partial and specific source information as conducted by Dodson et al. (1998), provides another future direction to examine how recollection of pictures and sounds may differ on different degrees of detail and specificity.

In conclusion, these results provide further demonstrations of the recognition memory advantage of pictures over environmental sounds and support for fuzzy-trace theory (Brainerd & Reyna, 2002), by showing that the recognition memory advantage for pictures is due to greater retrieval of both gist and perceptual information. These findings also provide further support for the label-feedback hypothesis (Lupyan, 2008). Finally, we provide strong evidence that both conceptual and perceptual distinctiveness can explain the higher recognition memory for pictures compared with sounds. It is important to note that we cannot state what characteristics of gist and verbatim representations, as predicted by conceptual–perceptual distinctiveness theory, differentiate them from representations predicted by other theories. In fact, perceptual distinctiveness and dual-coding theory would predict a stronger trace in memory for pictures than sounds and this trace would be similar to a verbatim representation predicted in fuzzy-trace theory, whereas a weaker trace in memory for sounds would be similar to gist representation predicted by fuzzy-trace theory. Thus, the specific type of memory trace would be different based on the theory. That is, the specific characteristics of the gist and verbatim representations would differ by the theory.

Future research could examine ways to mitigate the recognition memory for pictures compared with sounds by identifying distinctive features of sounds that make them memorable and providing a context for encoding of sounds that is similar to that of pictures. Another possible way to reduce or eliminate the advantage for pictures would be an implementation of a repetition manipulation with objects to test visual and auditory recognition, as Parks and Werner (2020) showed that repetition affected both auditory and visual recognition of words in similar ways.

## Supplemental Material

sj-docx-1-qjp-10.1177_17470218231202986 – Supplemental material for A conceptual–perceptual distinctiveness processing account of the superior recognition memory of pictures over environmental sounds Supplemental material, sj-docx-1-qjp-10.1177_17470218231202986 for A conceptual–perceptual distinctiveness processing account of the superior recognition memory of pictures over environmental sounds by Fahad N Ahmad, Savannah Tremblay, Michael D Karkuszewski, Marium Alvi and William E Hockley in Quarterly Journal of Experimental Psychology

## Notes

1. Note that we extend the application of the picture superiority effect (PSE), traditionally observed as a recognition advantage for pictures over words, to include pictures having a recognition advantage over sounds.

2. Using the report by Gloede and Gregg (2019) of high effect sizes and as guidelines indicate that partial eta squared is considered large at .14, we suggest that the effect in Gloede and Gregg (2019) could have been overestimated, as is often the case for first studies.

3. We inputted each of the partial effect size (i.e., partial eta squared) values reported by Gloede and Gregg (2019) into G*Power. The inputted calculation parameters were as follows: an error probability of 0.05, a power level of 0.80, two groups, number of measurements being 5, and lowest correlation among repeated measures set to 0.012 and a nonsphericity correction value of 1. For the main effect of stimulus type, G power generated a sample size of 6 to 8 participants, indicating that 20 participants are more than adequate to find a main effect of stimulus type. Gloede and Gregg (2019) reported the interaction with *p*-value of .049 as significant, although there would be varied opinion on the reliability of this interaction. The partial effect size for the interaction was .15, leading to a sample size prediction of 14 participants.

4. There was no significant difference in similarity between our added set of pictures and sounds with that of set of pictures and sounds by Gloede and Gregg (2019). For only the added 10 exemplar foil pictures, analysis of data from Experiment 3B showed that mean similarity in the exemplar condition (*M* = 3.40, *SD* = 0.21) compared to the exemplar condition without the new pictures (*M* = 3.37, *SD* = 0.32), was the same. For only the added 10 exemplar foil sounds, analysis of data from Experiment 3B showed that mean similarity in the exemplar condition (*M* = 2.95, *SD* = 0.42) compared to the exemplar condition without new sounds (*M* = 2.91, *SD* = 0.31), was not different. For only the added 10 novel foil pictures, analysis of data from Experiment 3B showed that mean similarity of novel foil with target picture (*M* = 1.20, *SD* = 0.21) compared to novel condition without new pictures (*M* = 1.17, *SD* = 0.32) was the same. For only the added 10 novel foil sounds, mean similarity in novel condition (*M* = 1.40, *SD* = 0.28) compared to novel condition without the new sounds (*M* = 1.37, *SD* = 0.32), was the same.

5. The zoom conference session and the practice phase were meant to check that participants could see instructions, pictures on their computer screen and hear sounds. In the online experiment, our study and test instructions were presented in auditory format, to make sure participants were paying attention to study and test instructions. We also checked the data for each participant that was tested, and no participant showed lower than chance in accuracy for pictures and sounds. Finally, we also checked the progress of each participant’s completion of the online research study on Gorilla.sc. If a participant spent too much time on the study or test phase or completed the sessions rapidly, then we would do a double check of the data including the response times of their recognition judgements.

6. We acknowledge that sounds do have the property of spatial distribution as in the case of spatial location in the environment, but the sounds presented in our experiments did not have such a property.
