# Supplementary material for: A conceptual–perceptual distinctiveness processing account of the superior recognition memory of pictures over environmental sounds
Source: Q J Exp Psychol (Hove). 2023 Oct 17;77(7):1555–80. doi: 10.1177/17470218231202986 (PMC11181738; doi:10.1177/17470218231202986)
Supplement: sj-docx-1-qjp-10.1177_17470218231202986 – Supplemental material for A conceptual–perceptual distinctiveness processing account of the superior recognition memory of pictures over environmental sounds [file sj-docx-1-qjp-10.1177_17470218231202986.docx]

Supplementary Material for:

**A Conceptual-Perceptual Distinctiveness Processing Account of the Superior Recognition Memory of Pictures Over Environmental Sounds**

Ahmad, F. N. ^1^, Tremblay, S. ^2, 3^, Karkuszewski, M. D. ^4^, Alvi, M. ^5^ and Hockley, W. E. ^1^

^1^ Psychology Department, Wilfrid Laurier University

^2^ Psychology Department, University of Toronto

^3^ Rotman Research Institute at Baycrest

^4^ Psychology Department, Western University

^5^ Psychology Department, York University

Word count: 17,635

**Author Note**

The Data and Stimuli have been made publicly available on the Open Science Framework: https://osf.io/fz6tk/

This research was supported by a Discovery Grant from the National Science and Engineering Research Council of Canada (NSERC) awarded to WEH. Portions of findings were presented at the 61^st^ Annual Meeting in Virtual Psychonomics Conference, November, 2020 and in the Annual Meeting of the Canadian Society for Brain, Behavior and Cognitive Science (CSBBCS), June, 2021 by all Authors. We have no conflicts of interest to disclose.

Correspondence concerning this article should be addressed to Dr. Fahad N. Ahmad, Department of Psychology, Wilfrid Laurier University, 75 University Ave W, Waterloo, ON N2L 3C5, Email: [fahmad@wlu.ca](mailto:fahmad@wlu.ca)

**Supplementary Material 1**

**Exp 2A**

**Confidence.** Mean confidence judgments for correct responses are shown in Table 3. Four participants were not included in the analysis of proportion of correct confidence responses because they showed inconsistent correct confident responses for novel and exemplar pictures and three participants also showed inconsistent responses for novel and exemplar sounds.

A 2 (Stimulus Type: picture vs. sounds) × 3 (Test Probe: target, exemplar foil, novel foil) repeated measures ANOVA based on mean confidence judgments revealed a significant main effect of Stimulus Type, *F*(1, 16) = 14.86,  *MSE* = .076, *p*  = .001, *η_­­_^2^_p_*  = .482, indicating higher confidence was shown for pictures (*M* = 2.70) compared to sounds (*M* = 2.49). The main effect of Test Probe was also significant, *F*(2, 32) = 10.28, MSE = .103, *p* = .000, *η_­­_^2^_p_* = .391. Higher confidence was shown for Targets (*M* = 2.78, *S. E* = .037) compared to novel foils (*M* = 2.56, *S.E* = .083) as shown with mean difference = .219, *p* = .044 with lowest confidence for exemplar foils (*M* = 2.43, *S.E* = .088) as shown with mean difference = .350, *p* = .003. There was no significant interaction between stimulus type and test probe, *F*(2, 32) = .181, *MSE* = .046, *p* = .835, *η_­­_^2^_p_*  = .011.

**Exp 2B**

**Confidence.** Mean confidence judgments for correct responses are shown in Table 3. Mean confidence judgments for correct responses are shown in Supplementary material. A 2 (Stimulus Type: picture vs. sounds) × 3 (Test: target vs. exemplar foil vs. novel foil) repeated measures ANOVA based on the mean confidence judgments showed there was no significant main effect of Stimulus Type, *F*(1, 16) = .132,  *MSE* = .109, *p*  = .721, *η_­­_^2^_p_*  = .008. There was a significant main effect of Test Probe, *F*(2, 32) = 16.32, *MSE* = .118, *p* = .000, *η_­­_^2^_p_*  = .505. Pairwise comparison showed higher confidence was shown for Targets (*M* = .280, *S.E* = .035) compared to novel foils (*M* = 2.47, *S.E* = .109) with mean difference = .327, *p* = .011, and exemplar foils (*M* = 2.33, *S.E* = .104) with mean difference = .082, *p* < .001. However, similar confidence in correct responses was shown for novel foils and exemplar foils, mean difference = -.137, *p* = .195. There was no significant interaction between stimulus type and test probe, *F*(2, 32) = .484, *MSE =* .066, *p* = .620, *η_­­_^2^_p_*  = .029.

**Exp 3A**

**Table 7**

*Mean proportions of responses for each similarity rating (i.e., Very similar, similar, somewhat similar, not similar) in each condition (i.e., Exemplar foil, Novel foil). This proportion was calculated by dividing the count of responses within each similarity rating by the number of trials (i.e., 13). For example, participant 2129 provided 9 very similar (i.e., VS) responses to picture pairs in the exemplar condition (i.e., a picture and a similar picture). As a result, to get proportion of similar responses for VS, we divided 9 by 13.*

| **Exp 3A** | **Exemplar** | | | | **Novel** | | | |
| --- | --- | --- | --- | --- | --- | --- | --- | --- |
|  | **VS** | **S** | **SS** | **NS** | **VS** | **S** | **SS** | **NS** |
| **Picture** | .71 (.22) | .19 (.14) | .08 (.11) | .02 (.07) | .00 (.00) | .01 (.02) | .08 (.12) | .91 (.12) |
| **Sound** | .37 (.29) | .33 (.16) | .25 (.16) | .06 (.10) | .02 (.06) | .04 (.09) | .22 (.16) | .72 (.20) |
| **Exp 3B** | **VS** | **S** | **SS** | **NS** | **VS** | **S** | **SS** | **NS** |
| **Picture** | .561 | .223 | .103 | .003 | .011 | .008 | .073 | .896 |
| **Sound** | .323 | .354 | .158 | .065 | .027 | .023 | .196 | .738 |

**Table 8**

*Overall Mean similarity rating by category and function for pictures and sounds in the Exemplar and Novel conditions.*

| **Experiment** | **Stimulus Type** | **Exemplar condition** | **Novel condition** | **Exemplar**  **Condition** | **Novel**  **condition** |
| --- | --- | --- | --- | --- | --- |
|  |  | **Category** | | **Function** | |
| **Exp 3A** | **Picture** | 3.24 (1.17) | .47 (.90) | 3.51 (.91) | .30 (.66) |
|  | **Sound** | 2.93 (1.06) | .63 (1.29) | 2.3 (1.43) | .63 (1.29) |

**Mean similarity across similarity ratings by collapsing mean similarity ratings across Semantic feature.** A 2 (Stimulus type: Picture vs. Sounds) × 2 (Condition: Exemplar, Novel) x 2 (Semantic Feature: Category, Function) repeated measures ANOVA was conducted on proportions of mean ratings. **Table 8** displays the mean similarity ratings. There was no significant main effect of Stimulus Type, *F*(1, 19) = .205, *MSE* = .967, *p* =.656, *η_­­_^2^_p_*  = .011 and Semantic Feature, *F*(1, 19) = 2.40, *MSE* = 1.69, *p* = .138, *η_­­_^2^_p_*  = .112. There was a significant effect of condition, *F*(1, 19) = 145.92, *MSE* = 1.45, *p* = .000, *η_­­_^2^_p_*  = .885; as expected there were higher ratings of similarity in the exemplar than novel condition. Interestingly, there was an interaction between stimulus type and condition, *F*(1, 19) =17.48, *MSE* = 1.10, *p*  = .001, *η_­­_^2^_p_*  = .479. Mean similarity was higher in the exemplar (*M* = 3.38, *S.E* = .18) compared to the novel condition (*M* = .385, *S.E* = .129) for pictures compared to sounds. As confirmed by Bonferroni comparisons, the mean difference between mean similarity in exemplar and novel condition was higher for pictures (*M diff* = .764, *p* < .001) compared to sounds (*M diff* = .623, *p* =.034). There was a significant interaction between stimulus type and semantic feature, *F*(1, 19) = 10.07, *MSE* = .543, *p* = .005, *η_­­_^2^_p_*  = .346. There was no difference in similarity for pictures in terms of semantic feature, whereas for sounds, there was higher similarity based on category (*M* = 2.16, *S.E* = .17) than function (*M* = .147, *S.E* =.23).

**Table 9**

*Displayed are the Mean proportions of responses for each similar rating* *by Category and Function by stimulus type (i.e., pictures and sounds) in exemplar and novel condition. For each similarity rating, that is, very similar, similar, somewhat similar and not similar; a mean proportion of responses was calculated by dividing by the number of trials being thirteen trials. Abbreviations: VS-Very similar, S-Similar, SS-Somewhat similar, NS_Not similar.*

|  | **Exemplar** | | | | | | | | | | | | |
| --- | --- | --- | --- | --- | --- | --- | --- | --- | --- | --- | --- | --- | --- |
|  | **Category** | | | | **Function** | | | | **None** | | | | |
|  | **VS** | **S** | **SS** | **NS** | **VS** | **S** | **SS** | **NS** | **VS** | **S** | **SS** | **NS** |  |
| **Pictures** | 0.42 (.24) | .123 (.12) | .038 (.07) | .011  (.05) | 0.28 (.20) | .069 (.10) | .046 (.09) | .00  (.00) | .00 (.00) | .0038 (.07) | .00 (.00) | .004 (.017) |  |
| **Sounds** | .30 (.19) | .21  (.15) | .14 (.16) | .008 (.007) | .07 (.10) | .11 (.17) | .06 (.09) | .00  (.00) | .00 (.00) | .003 (.017) | .046  (.068) | .038  (.052) |  |

|  | **Novel** | | | | | | | | | | | | |
| --- | --- | --- | --- | --- | --- | --- | --- | --- | --- | --- | --- | --- | --- |
|  | **Category** | | | | **Function** | | | | **None** | | | | |
|  | **VS** | **S** | **SS** | **NS** | **VS** | **S** | **SS** | **NS** | **VS** | **S** | **SS** | **NS** |  |
| **Pictures** | .00 (.00) | .008 (.00) | .011 (.11) | .019 (.05) | .00 (.00) | .00 (.00) | .007 (.0236) | .011 (.037) | .00 (.00) | .00 (.00) | .06 (.11) | .88 (.15) |  |
| **Sounds** | .011 (.04) | .023 (.071) | .077 (.079) | .046 (.14) | .011 (.04) | .00 (.00) | .011 (.04) | .011 (.037) | .00 (.00) | .011 (.028) | .13 (.162) | .66 (.25) |  |

**Analysis of similarity based Semantic features***.* A 2 (Stimulus type: Picture, Sounds) × 2(Semantic feature: Category, Function) x 2 (Condition: Exemplar, Novel) x 4 (Level of similarity: Very similar, Similar, Somewhat similar, Not similar) repeated measures ANOVA was conducted on proportion of similar responses. **Table 9** contains the relevant means. There was no significant effect of Stimulus type, *F*(1, 19) = 1.57, *MSE* = .001, *p* =.226, *η_­­_^2^_p_*  = .076. There was a significant main effect of semantic feature, *F*(1, 19) = 7.64, *MSE* =.048, *p* =.012, *η^2^_p_* = .287. Category was chosen significantly more (*M* = .091, *S.E* = .010) than function (*M* = .044, *S.E* = .008) as a basis of similarity rating. There was a significant main effect of condition, *F*(1, 19) = 475.71, *MSE* = .004, *p* < .001, *η_­­_^2^_p_*  = .962. There were higher mean similarity responses in exemplar (*M* = .119, *S.E* = .001) than in novel condition (*M* = .016, *S.E* = .004). There was a significant main effect of similarity rating, *F*(3, 57) = 31.56, *MSE* =.014, *p* =.000, *η_­­_^2^_p_* = .624. Highest category and function responses were provided with the higher the level of similarity.

There was a significant interaction between stimulus type and level of similarity, *F*(3, 57) = 20.93, *MSE* = .006, *p* < .001, *η_­­_^2^_p_* = .518. For pictures, there was higher proportion of similar responses in very similar level of similarity (*M* = .177, *S.E* = .012), whereas, for sounds there was higher proportion of similar responses in similar and somewhat similar level of similarity (*M* =.01, *S.E* =.011; *M* = .069, *S.E* =.011). Moreover, there was a significant interaction between stimulus type and semantic feature, *F*(1, 19) = 16.6, *MSE* = .004, *p* =.001, *η_­­_^2^_p_* = .466. There was lower similarity rating for pictures (*M* = .080, *S.E* = .009) than for sounds (*M* = .103, *S.E* = .011) in terms of Category as shown by Bonferroni comparison (mean diff = .023, *p* = .000). However, based on Function, there was higher similarity rating for pictures (*M* =.052, *S.E* = .008) compared to sounds (*M* = .035, *S.E* = .0009), as shown by Bonferroni comparison (mean diff = .017, *p* = .012)

There was also a significant interaction between stimulus type and condition, *F*(1, 19) = 45.02, *MSE* = .001, *p* = .000, *η_­­_^2^_p_* = .703. There was higher similarity rating in the exemplar condition for pictures (*M* = .125, *S.E* = .000) compared to sounds (*M* = .114, *S.E* = .002), supported by Bonferroni comparison (mean diff = .011, *p* =.000). Conversely, there was lower similarity rating in the novel condition for pictures (*M* =.007, *S.E* = .003) compared to sounds (*M* =.024, *S.E* =.006), supported by Bonferroni comparison (mean diff = .017, *p* = .001).

There was a significant interaction between condition and level of similarity, *F*(3, 57) = 53.8, *MSE* = .011, *p* < .001, *η_­­_^2^_p_* = .739. For the exemplar condition, there was highest proportion of similar responses in the very similar level of similarity (*M* =.2.70, *S.E* = .021), whereas for novel condition, the highest proportion of similar responses was in the somewhat similar (*M* = .024, *S.E* =.005) and high level of similarity (*M* = .022, *S.E* =.014).

**Mean similarity across similarity ratings by collapsing mean similarity ratings across Perceptual feature.** A 2 (Condition: Exemplar, Novel) x 2 (Perceptual Feature: Colour, Shape) repeated measures ANOVA was conducted on overall similar ratings for pictures. Relevant means are in **Table 10**.

**Table 10**

*Overall Mean similarity rating by perceptual feature for pictures and sounds in the Exemplar and Novel condition.*

| **Experiment 3A** | | |
| --- | --- | --- |
| ***Picture*** | ***Exemplar*** | ***Novel*** |
| **Colour** | 3.58 (.94) | .72 (.99) |
| **Shape** | 3.56 (.66) | .05 (.22) |
| ***Sound*** | ***Exemplar*** | ***Novel*** |
| **Loudness** | 2.72 (1.30) | .82 (1.16) |
| **Pattern** | 3.17 (.90) | 1.56 (1.10) |
| **Pitch** | 2.84 (.82) | 1.13 (1.02) |

There was a significant main effect of Condition, *F*(1, 19) = 298.37, *MSE* = .68, *p* < .001, *η_­­_^2^_p_*  = .940. As expected, there was higher mean similarity rating in the exemplar (*M* = 3.57, *S.E* = .148) than in the novel condition (*M* = .388, *S.E* = .115). There was a strong trend for a significant main effect of Perceptual feature, *F*(1, 19) = 3.73, *MSE* = 2.45, *p* = .068, *η_­­_^2^_p_* = .164. There was a significant interaction between condition and perceptual feature, *F*(1, 19) = 2.13, *MSE* = .304, *p* = .016, *η_­­_^2^_p_* = .270. There was higher mean similarity based on colour (*M* = 3.58, *S.E* = .212) and shape (*M* =.356, *S.E* =.147) in the exemplar condition compared to the novel condition.

A 2 (Condition: Exemplar, Novel) x 2 (Perceptual Feature: Loudness, Pattern, Pitch) repeated measures ANOVA was conducted on proportions of mean ratings for sounds. There was a significant main effect of condition, *F*(1, 19) = 51.8, *MSE* = 1.74, *p* < .001, *η_­­_^2^_p_* = .732. As expected, there was a higher mean similarity rating based on perceptual feature, in the exemplar (*M* = 2.91, *S.E* = .15) than in the novel (*M* = 1.18, *S.E* = .18) condition. There was a also a significant main effect of perceptual feature, *F*(2, 38) = 3.7, *MSE* = .77, *p* = .014, *η_­­_^2^_p_* = .201. Similarity rating was based more on pattern (*M* =2.37, *S.E* = .13) than loudness (*M* = 1.77, *S.E* =.19). Bonferroni comparison was significant (*p* =.009, *mean diff* = .598).

**Table 11**

*Displayed are the Mean proportions of responses for each similar rating by Colour and Shape for pictures and loudness, pattern, and pitch by stimulus type (i.e., pictures and sounds) in exemplar and novel condition. For each similarity rating, that is, very similar, similar, somewhat similar, and not similar; a mean proportion of responses was calculated by dividing by the number of trials. Abbreviations*: VS-Very similar, S-Similar, SS-Somewhat similar, NS_Not similar, Cat-Category, Func-Function

|  | **Colour** | | | | **Shape** | | | | **None** | | | |
| --- | --- | --- | --- | --- | --- | --- | --- | --- | --- | --- | --- | --- |
| **Pictures** | **VS** | **S** | **SS** | **NS** | **VS** | **S** | **SS** | **NS** | **VS** | **S** | **SS** | **NS** |
| **Exemplar** | 0.16 | 0.046 | .00 | 0.011 | 0.53 | .134 | .042 | .00 | 0.01 | .011 | .034 | 0.003 |
| **Novel** | .00 | 0.00 | .05 | 0.02 | .00 | .00 | .00 | .00 | .00 | .00 | 0.015 | 0.88 |

|  | **Loudness** | | | | **Pattern** | | | | **Pitch** | | | | | **None** | | | |
| --- | --- | --- | --- | --- | --- | --- | --- | --- | --- | --- | --- | --- | --- | --- | --- | --- | --- |
| **Sounds** | **VS** | **S** | **SS** | **NS** | **VS** | **S** | **SS** | **NS** | **VS** | **S** | **SS** | **NS** | **VS** | | **S** | **SS** | **NS** |
| **Exemplar** | 0.08 | 0.09 | 0.03 | .00 | 0.19 | 0.119 | 0.05 | 0 | 0.1 | 0.10 | 0.1 | 0.008 | 0.003 | | 0.019 | 0.06 | 0.04 |
| **Novel** | 0.008 | 0 | 0.05 | 0.023 | 0.0115 | 0.00 | 0.07 | 0.011 | 0 | 0.008 | 0.06 | 0.04 | 0.004 | | 0.023 | 0.04 | 0.65 |

**Analysis of similarity based on Perceptual features***.* We analyzed separately the difference in similarity rating based on perceptual feature for pictures and sounds, as there were different perceptual features used for rating of similarity of pictures and sounds.

A 2(Perceptual feature: Colour, Shape) x 2 (Condition: Exemplar, Novel) x 4 (Level of similarity: Very similar, Similar, Somewhat similar, Not similar) repeated measures ANOVA was conducted on mean proportions of responses for each similar rating for **pictures**. Table 10 contains the relevant means.

There was a significant main effect of perceptual feature, *F*(1, 19) = 13.90, *MSE* = .015, *p* = .001, *η_­­_^2^_p_* = .422. There was a lower mean similarity rating for pictures based on colour (*M* =. 038, *S.E* =.011) than shape (*M* =.090, *S.E* = .006). There was a significant main effect of condition, *F*(1, 19) = 410.8, *MSE* = .000, *p* < .001, *η_­­_^2^_p_* = .956. There was higher mean similarity rating for pictures in exemplar (*M* = .117, *S.E* = .002) than novel (M = .011, S.E = .004). There was a main effect of Level of Similarity, *F*(3, 57) = 55.87, *MSE* = .008, *p* <.001, *η_­­_^2^_p_* = .746. Highest proportion of similarity responses were in very similar level of similarity. Moreover, there was an interaction between perceptual feature and condition, *F*(1, 19) = 41.29, *MSE* = ..009, *p* <.001, *η_­­_^2^_p_* = .685. For perceptual feature of colour, higher proportion of similarity rating responses were provided in exemplar (*M* = .057, *S.E* = .012) compared to the novel condition (*M* = .020, *S.E* = .007) as supported by Bonferroni comparison (mean diff =.037, *p* =.004). Moreover, for category, there was higher proportion of similarity rating responses provided in exemplar (*M* = .18, *S. E* = .012) compared to novel condition (*M* = .002, *S.E* = .02) as supported by Bonferroni comparison (mean diff = .178, *p* < .001).

There was an interaction between condition and level of similarity, *F*(3, 57) = 83.8, *MSE* = .007, *p* <.001, *η_­­_^2^_p_* = .815. Proportion of similar responses was higher in very similar level of similarity (*M* = .348, *S.E* = .024) compared to other levels of similarity in the exemplar foil condition. In contrast, proportion of not very (*M* = .015, *S.E* = .009) and not similar responses (*M* = .025, *S.E* = .012) were highest in novel foil condition. There was also an interaction between Perceptual feature, Condition and Level of similarity, *F*(3, 57) = 16.99, *MSE* = .008, *p* < .001, *η_­­_^2^_p_* = .472. As there were several significant effects, the main analyses accounting for the interaction of the three variables will be provided here. Post-hoc Bonferroni comparisons showed the mean for very similar responses based on colour to pictures in exemplar condition (*M* = 0.162, *S.E* = 0.027) was lower than very similar responses based on shape to pictures in exemplar condition (*M* = 0.535, *S.E* = 0.049), mean diff = .373, *t*(19) = 5.86, *p* <.001. Similarly, the mean for similar responses based on colour to pictures in exemplar condition (*M* = 0.046, *S.E* = 0.025) was lower than mean for similar responses based on shape (*M* = 0.135, *S.E* = 0.049), mean diff = .09, *t*(19) =2.26, *p* =.036). As expected, mean of very similar responses in exemplar condition were higher than in novel condition. For example, mean similar responses to pictures in exemplar condition based on colour (*M* = 0.162, *S.E* = 0.027) was higher than mean similar responses to pictures in the novel condition, where colour was the chosen feature (*M* = 0.008, *S.E* = 0.005), mean diff = .161, *t*(19) = 6.05, *p* < .001.

A 3(Perceptual feature: Loudness, Pitch, Pattern) x 2 (Condition: Exemplar, Novel) x 4 (Level of similarity: Very similar, Similar, Somewhat similar, Not similar) repeated measures ANOVA was conducted on Mean proportions of responses for each similar rating for **sounds**. Table 10 contains the relevant means. There was no significant main effect of perceptual feature, *F*(2, 38) = 2.53, MSE = .008, *p* =.093, *η_­­_^2^_p_* = .118. Similar to pictures, there was a significant main effect of condition, *F*(1, 19) = 123.6, MSE = .002, *p* < .001, *η_­­_^2^_p_* = .867. As found with pictures, there was higher similarity responses were provided in exemplar (*M* =. 073, *S.E* =.002) than novel condition (*M* = .025, *S.E* = .005). As found with pictures, there was also a significant main effect of Level of similarity, *F*(3, 57) = 8.6, *MSE* = .008, *p* < .001, *η_­­_^2^_p_* = .311. Highest proportion of similarity responses was in the highest level of similarity (*M* = .065, *S.E* = .008) compared to lowest level of similarity (*M* =.014, *S.E* = .007). In contrast to pictures, there was no interaction between perceptual feature and condition, *F*(2, 38) = 2.34, *MSE* = .005, *p* = .109, *η_­­_^2^_p_* = .110.

As found with pictures, there was an interaction between condition and level of similarity, *F*(3, 57) = 17.6, *MSE* = .008, *p* < .001, *η_­­_^2^_p_* = .481. In the exemplar condition, highest proportion of similarity responses were shown in very similar level of similarity (*M* = .123, *S.E* = .015) compared to lowest level of similarity (*M* = .003, *S.E* = .003) as supported by Bonferroni comparison (mean diff = .121, *p* <.001). In contrast, in the novel condition, highest proportion of similarity responses were shown in the somewhat similar (*M* = .057, *S.E* = .011) compared to very similar level of similarity (*M* = .006, *S.E* = .004).

There was an interaction between perceptual feature and level of similarity, *F*(6, 114) = 3.00, *MSE* = .004, *p* = .009, *η_­­_^2^_p_* = .137. In terms of loudness, there were more similar responses for very similar level of similarity compared to not similar level of similarity as supported by Bonferroni comparison (mean diff = .033, *p* = .035). Whereas in terms of pitch, the proportion of similar responses were higher in somewhat similar compared to not similar level of similarity (mean diff = .054, *p* = .009). Interestingly, in terms of pattern, proportion of similar responses were significantly higher for all three levels of similarity compared to the not similar level of similarity as supported by Bonferroni comparisons (mean diff = .016, *p* = .000; mean diff = .011, *p* = .000; mean diff = .006, *p* = .006). Finally, there was an interaction between perceptual feature, condition and level of similarity, *F*(6, 114) = 2.39, *MSE* = .005, *p* = .03, *η_­­_^2^_p_* =.112. As there were several significant effects, the main analyses represented in the Bonferroni post-hoc comparisons accounting for the interaction of the three variables will be provided here. There was significantly higher very similar responses based on pattern to sounds (*M* = 0.188, *S.E* = 0.034) compared to very similar responses based on loudness (*M* = 0.081, *S.E* = 0.018) in the exemplar condition, *t*(19)=4.86, *Mean diff* = 0.108, *p* < .001. Conversely, there was significantly lower very similar responses based on pitch (*M* = 0.100, *S.E* = 0.020) compared to very similar responses based on pattern (*M* = 0.188, *S.E* = 0.034) in the exemplar condition, *t*(19) = 3.99, *Mean diff* = 0.088, *p* = 0.023. Mean very similar responses based on pattern to sounds in exemplar condition (*M* = 0.188, *S.E* = 0.034) was higher than in novel condition (*M* = 0.012, *S.E* = 0.008), *t*(19) = 7.73, *Mean diff* = 0.073, *p* < .001 .

**Supplementary Material 2**

**Experiment 3A**

| **Sound test trial** | **Exemplar** | Drum Roll | Drum Roll |
| --- | --- | --- | --- |
|  | **Exemplar** | Music Box | Music Box |
|  | **Exemplar** | Piano | Piano |
|  | **Exemplar** | Police Siren | Police Siren |
|  | **Exemplar** | Rain | Rain |
|  | **Exemplar** | Ship | Ship |
|  | **Exemplar** | Train | Train |
|  | **Exemplar** | Windchimes | Windchimes |
|  | **Exmplar** | Auto racing | Auto racing |
|  | **Exemplar** | Clap | Clap |
|  | **Exemplar** | Bowling | Bowling |
|  | **Exemplar** | Bubbles | Bubbles |
|  | **Novel** | Kettle | Horse |
|  | **Novel** | Latex Gloves | Helicopter |
|  | **Novel** | Frogs | Match |
|  | **Novel** | Footsteps | Microwave |
|  | **Novel** | Cutlery | Owl |
|  | **Novel** | Crying Baby | Pill Bottle |
|  | **Novel** | Rollercoaster | Ice in a Glass |
|  | **Novel** | Sharpening Knives | Toilet |
|  | **Novel** | Chopping Vegetables | Basketball Swish |
|  | **Novel** | Beads | Turning Pages |
|  | **Novel** | Soccer Kick | Coins |
|  | **Novel** | Uncorked Wine | Cat |
|  | **Novel** | Zooming in and out | Leaves Crunching |
| **Picture test trial** | **Exemplar** | Airplane | Airplane |
|  | **Exemplar** | Alarm Bell | Alarm Bell |
|  | **Exemplar** | Dog Barking | Dog Barking |
|  | **Exemplar** | Bird | Bird |
|  | **Exemplar** | Camera | Camera |
|  | **Exemplar** | Cellphone | Cellphone |
|  | **Exemplar** | Chickens | Chickens |
|  | **Exemplar** | Chimp | Chimp |
|  | **Exemplar** | Motorcycle | Motorcycle |
|  | **Exemplar** | Ping pong | Ping pong |
|  | **Exemplar** | Ticktock | Ticktock |
|  | **Exemplar** | Rocking Chair | Rocking Chair |
|  | **Exemplar** | Trumpet | Trumpet |
|  | **Novel** | Heartbeat | Blow dryer |
|  | **Novel** | Drill | Brushing Teeth |
|  | **Novel** | Mosquito | Cheer |
|  | **Novel** | Fire | Curtain |
|  | **Novel** | Basketball dribble | Dishes Breaking |
|  | **Novel** | Electric Grater | Deep Breathing |
|  | **Novel** | Water Splash | Frying Pan |
|  | **Novel** | Lasers | Garbage Disposal |
|  | **Novel** | Sheep | Waves |
|  | **Novel** | Shopping Cart | Door |
|  | **Novel** | Laugh | Pouring Tea |
|  | **Novel** | Bicycle | Vacuum |
|  | **Novel** | Cereal | Baseball |

**Table 1.** List of stimuli and verbal labels presented in a counterbalanced condition of Experiment 3A. Verbal labels were presented for both pictures and sounds. Stimuli and Conditions were presented in randomized order.
